# A nanofluidic device for parallel single nanoparticle catalysis in solution

**DOI:** 10.1038/s41467-019-12458-1

**Published:** 2019-09-27

**Authors:** Sune Levin, Joachim Fritzsche, Sara Nilsson, August Runemark, Bhausaheb Dhokale, Henrik Ström, Henrik Sundén, Christoph Langhammer, Fredrik Westerlund

**Affiliations:** 1 Department of Biology and Biological Engineering, Chalmers University of Technology, SE-412 96 Gothenburg, Sweden; 2 Department of Physics, Chalmers University of Technology, SE-412 96 Gothenburg, Sweden; 3 Department of Chemistry and Chemical Engineering, Chalmers University of Technology, SE-412 96 Gothenburg, Sweden; 4 Department of Mechanics and Maritime Sciences, Chalmers University of Technology, SE-412 96 Gothenburg, Sweden

**Keywords:** Catalysis, Nanoscale devices, Chemical physics

## Abstract

Studying single catalyst nanoparticles, during reaction, eliminates averaging effects that are an inherent limitation of ensemble experiments. It enables establishing structure–function correlations beyond averaged properties by including particle-specific descriptors such as defects, chemical heterogeneity and microstructure. Driven by these prospects, several single particle catalysis concepts have been implemented. However, they all have limitations such as low throughput, or that they require very low reactant concentrations and/or reaction rates. In response, we present a nanofluidic device for highly parallelized single nanoparticle catalysis in solution, based on fluorescence microscopy. Our device enables parallel scrutiny of tens of single nanoparticles, each isolated inside its own nanofluidic channel, and at tunable reaction conditions, ranging from the fully mass transport limited regime to the surface reaction limited regime. In a wider perspective, our concept provides a versatile platform for highly parallelized single particle catalysis in solution and constitutes a promising application area for nanofluidics.

## Abstract (web summary)

Single nanoparticle catalysis studies enable structure–function correlations beyond averaged properties. Here, the authors present a versatile nanofluidics- and fluorescence-based platform for highly parallelized single particle catalysis in solution.

## Introduction

Nanoparticles are used extensively in catalysis due to their specific activity and relative abundance of highly active sites^1–3^. In this respect, great progress has been made in establishing structure–activity correlations^4–8^. Nevertheless, to date most nanoparticle catalysis studies are performed on ensembles containing thousands to billions of particles. Hence, the data obtained generally describe the response of an average nanoparticle. Additionally, nanoparticles are structurally heterogeneous at the atomic level^9–11^, and two nominally identical particles from the same synthesis batch may exhibit substantially different activity or selectivity^12,13^. Therefore, measurements on single nanoparticles are essential to uncover particle specific properties. In response, experimental methods that enable studies of catalytic processes on individual nanoparticles are emerging^3,14–17^. The most prominent methods for single particle catalysis in solution are single-molecule fluorescence microscopy (SMFM)^4,12,18,19^, surface-enhanced raman scattering (SERS)^20,21^, tip-enhanced raman spectroscopy (TERS)^22,23^, scan electrochemical microscopy (SECM)^24^, synchrotron-radiation-based infrared nanospectroscopy (SINS)^6^ and surface plasmon spectroscopy (SPS)^25^. Even though these are all effective techniques, they have limitations, such as that they require very low reactant concentrations and/or reaction rates^14^, that they have low throughput^12^, or that they probe only one particle at a time.

In this paper, we present a nanofluidic device that, in combination with epifluorescence microscopy, enables simultaneous and parallel scrutiny of catalyst activity in the liquid phase on several tens of catalyst nanoparticles, each isolated in its own nanofluidic channel. These nanochannels enable laminar flow of reactants to, and product molecules from, the individual particles and retain the fluorescent molecules within the focal plane of the microscope. In this way our approach enables parallel scrutiny of individual catalyst nanoparticles under a wide range of conditions. This is in stark contrast to single-molecule fluorescence microscopy, where reactants need to be highly diluted and experiments are carried out at—from an applications point of view—unrealistically low reactant concentrations^4,12,18,19^. For example, the transition between the mass transport and surface reaction limited regimes of a catalyst located in a nanoconfined volume can be visualized at the single nanoparticle level and analyzed as a function of nanoparticle size. This is important for understanding catalysis, since the overall activity of the system is dictated by the interplay between transport of reactants to the particle surface and the rate-limiting elementary step of the surface reaction, which in turn may be controlled by particle size and structure^26^. To this end, the nanofluidic design of our device also ensures that the reaction conditions are identical for all the catalyst particles in the device, and that up- or downstream conversion effects by neighboring particles can be completely eliminated, since each nanoparticle is isolated inside its own nanochannel (Supplementary Note 1). Using this platform and the catalytic reduction of fluorescein by borohydride over Au nanoparticles^27^ as model reaction, we show that in contrast to other single particle approaches^4,12,17–19^, an averaged turnover frequency (ToF) of ca. 0.1 s^−1^ per site can be reached, which is in the range of relevant industrial applications^28^. Additionally, we can probe the same nanoparticle in situ at reactant concentrations ranging from a situation where the reaction rate is fully mass transport limited to the surface reaction controlled regime.

## Results

### Layout of the nanofluidic device

**Fig. 1:**
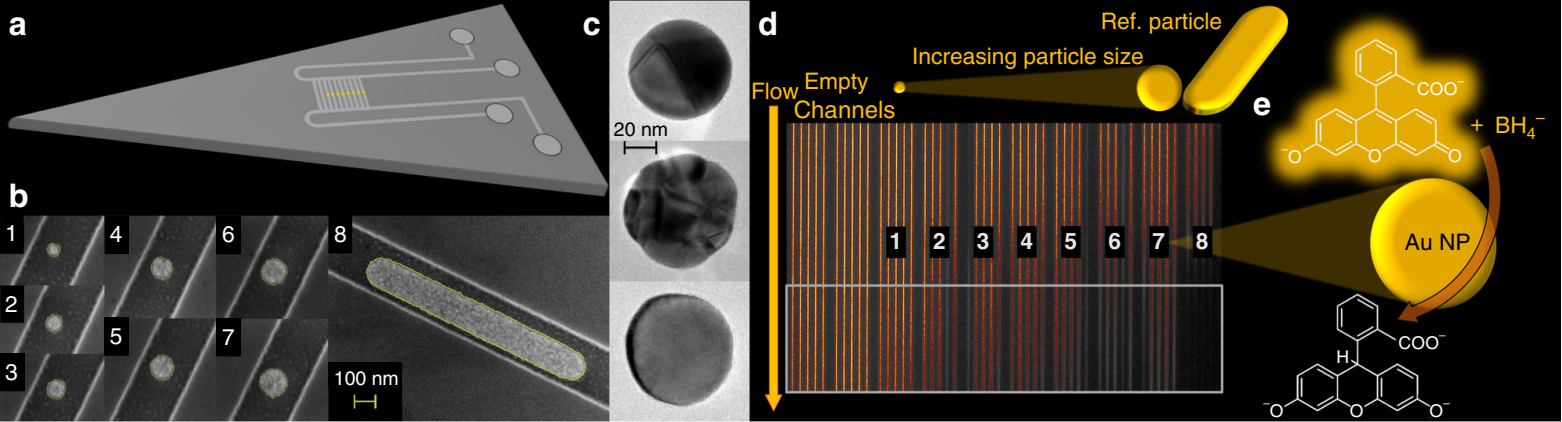
Representation of the experimental setup and the studied reaction. **a** Schematic depiction of the nanofluidic chip comprised of microchannels with macroscopic inlets that contact an array of two identical sets of 55 nanofluidic channels, each decorated with a single Au nanoparticle in the center. The nanochannels are 100 nm high, 250 nm wide, and 350 µm long. **b** Representative scanning electron microscopy (SEM) images of Au nanoparticles with systematically increasing sizes, taken inside a nanochannel prior to bonding of the glass lid: (1) 64 nm, (2) 82 nm, (3) 91 nm, (4) 102 nm, (5) 113 nm, (6) 121 nm, (7) 128 nm, and (8) patch of 139 × 1138 nm. **c** Transmission electron microscopy (TEM) images of 3 representative Au nanoparticles (~66 nm in size) after annealing (mimicking the conditions of lid bonding during assembly of the nanofluidic device) and cleaning in a solution of 4.3 wt% ammonia and 4.3 wt% hydrogen peroxide in milli-Q water. Note the distinct structural differences in terms of grain structure and grain size, despite the essentially identical dimensions. **d** Fluorescence image of an array of 50 nanochannels (an additional set of empty reference channels is not shown). Each set of 5 channels with a number label is decorated with nominally identically sized (same as **b**) nanoparticles. The ten empty channels to the left serve as negative control. Note the decreased fluorescence intensity downstream of the catalyst particle position and how it varies at the single nanoparticle level. Three channels were clogged from the start (dark channels at nanoparticle sizes 2, 5, and 6) and were not evaluated. **e** Schematic of the reduction of fluorescein on an Au nanoparticle catalyst, which renders the product molecule non-fluorescent and thus generates the optical contrast for analysis

At the chip level, the overall design is such that in- and outlet microchannels contact an array of nanochannels, which host single Au nanoparticles with well-defined dimensions (Fig. 1a and Supplementary Fig. 1). The nanochannel array is comprised of 11 sets of 5 nanochannels (100 nm high, 250 nm wide and 350 µm long), each decorated with a set of single Au particles with nominally identical size. Three 5-channel sets are kept empty to enable simultaneous negative control experiments on the same chip.

At the heart of our nanofluidic device are the catalytic Au nanoparticles that we have grown with high precision inside their corresponding individual nanochannels (Fig. 1b) by means of nanofabrication based on electron-beam lithography (Supplementary Note 1). All particles were grown simultaneously to a thickness of 20 nm through Au evaporation and ranged in size from 64 nm to 128 nm in diameter (Fig. 1b). Furthermore, we fabricated an elongated Au patch (139 × 1138 nm) and used it as a reference, mimicking an infinite system. As apparent from the scanning electron microscopy (SEM) images in Fig. 1b, directly after Au evaporation the nanoparticles exhibit a rough surface structure and are characterized by very small grains. Since it is impossible to structurally characterize the nanoparticles in detail after the complete assembly of the nanofluidic chip due to the anodically bonded glass lid (a step that requires 550 °C and thus is expected to significantly alter particle microstructure), we carried out a detailed characterization of a set of 66 nm particles on an open surface analog. For this purpose, we exposed these particles to the same sequence of temperature treatment and chemical cleaning steps as the particles inside the nanochannels experience during chip assembly and reaction. Evidently, the obtained nanoparticles vary slightly in size (65.8 ± 1 nm in diameter) and considerably in microstructure, ranging from single-crystalline to polycrystalline with up to approximately 10 grains (Fig. 1c and Supplementary Figs. 2–4). These microstructure differences originate from the annealing processing step (mimicking the anodic bonding of the lid), during which the particles recrystallize and attain a more thermodynamically stable structure with predominantly low index facets. The abundance, size, and distribution of facets are, however, expected to vary extensively between the individual particles due to the significantly different number of grains (Fig. 1c and Supplementary Fig. 2)^29^. To indirectly confirm this particle-to-particle heterogeneity for the actual Au nanoparticles inside the sealed nanofluidic channels, we measured their optical dark-field scattering spectra, which indeed exhibit different intensities and plasmon resonance frequencies (Supplementary Figs. 5 and 6), indicative of structural heterogeneity^30^.

Proceeding our discussion to the catalytic reaction, Fig. 1d depicts a CCD image of a nanochannel array, where the brightness of each channel corresponds to the fluorescence emission from fluorescein flowing through the fluidic system together with sodium borohydride in milli-Q water. The numbered labels indicate the position of the Au nanoparticles in the channel and refer to the specific particle size. Evidently, in channels decorated with an Au nanoparticle, the emission intensity is reduced downstream of the particle. This observation is in good agreement with an Au-catalyzed reduction of fluorescein with NaBH_4_ as the reduced form of fluorescein is non-fluorescent (Fig. 1e)^27^. The reaction product was analyzed in detail and verified by performing a batch reaction followed by detailed characterization using nuclear magnetic resonance spectroscopy (NMR), high resolution mass spectrometry (HRMS) and infrared spectroscopy (IR) (Supplementary Note 3, Supplementary Figs. 7–9).

### Reactant intensity profile in a nanochannel

**Fig. 2:**
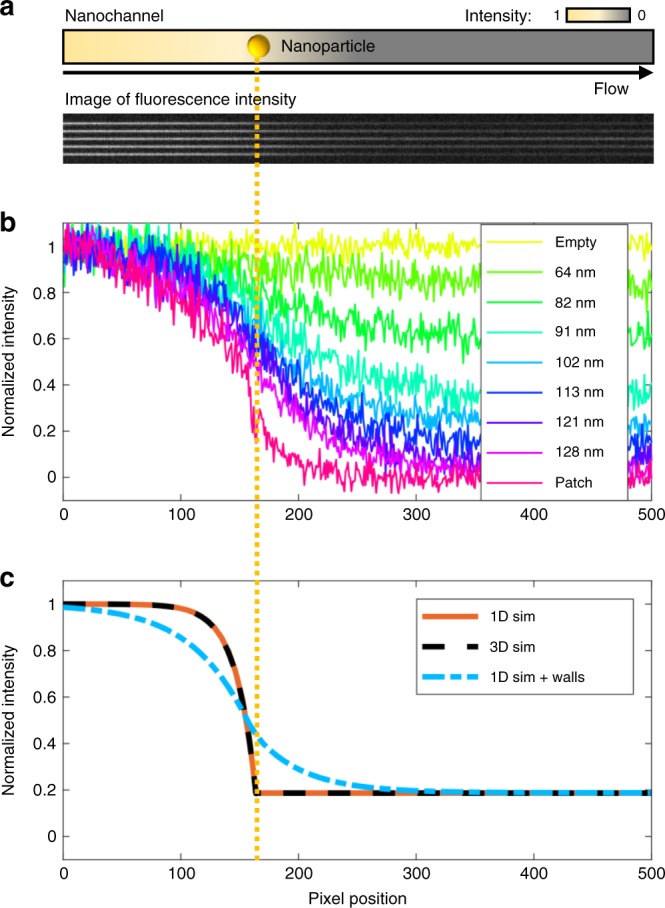
Fluorescence intensity distributions during reaction. **a** Schematic depiction of the fluorescence intensity distribution along a nanochannel containing a nanoparticle at the marked position together with a corresponding real fluorescence image of a set of five nanochannels, each containing a nanoparticle with a diameter of 121 nm. Note the decrease in fluorescence intensity downstream of the nanoparticles. **b** Normalized, with respect to the average of all empty reference channels, experimentally measured, fluorescence intensity profiles along different sets of nanochannels, depicted as the mean value of 5 channels for each particle size. For all channels, the catalyst particle is located at pixel position 164 (yellow dotted line). **c** Fluorescence intensity profiles simulated in 1D (orange line) and in 3D (dashed black line), as well as in 1D with wall adsorption, desorption, and diffusion (blue dash-dotted line)

For further quantitative analysis of the reaction in the nanochannels, we integrated the fluorescence intensity in each nanochannel within the area highlighted by the dashed rectangle in Fig. 1d to ultimately derive the reaction rate at each single nanoparticle (Supplementary Fig. 10). First, however, it is interesting to analyze the corresponding characteristics of the fluorescence intensity profile, that is, the fluorescein concentration profile (Supplementary Fig. 12b), along a nanochannel after reactant injection. This is uniquely facilitated by the nanofluidics approach used, since it confines the fluorophores in the microscope focal plane along the entire channel throughout the experiment. Such fluorescence intensity profiles are shown as the mean of all five particles investigated for each size in Fig. 2a. Clearly, the intensity decreases gradually along the channel and the profile exhibits an inverted sigmoidal shape with the steepest slope around the position of the nanoparticle for all sizes. This shape becomes more pronounced as the incoming fluorescein concentration is reduced during the course of the experiment (Supplementary Figs. 11 and 12). Interestingly, however, the fluorescence intensity also decreases after the particle where the reaction occurs. This is surprising at first, and to understand this behavior we resort to 1D and 3D simulations (that are in quantitative agreement) of our system for two different scenarios (Fig. 2b and Supplementary Figs. 13 and 14). The first one, under the assumption that reaction at the particle position and diffusion are the only relevant events, yields a fluorescence profile that steeply decreases at the catalyst particle position, as well as shows certain depletion upstream of the particle due to diffusion. Downstream of the catalyst, the profile is constant. However, the obtained profiles for 1D and 3D are significantly different from the experimentally observed ones downstream of the nanoparticle, where they do not capture the continued fluorescence intensity decrease. This implies a change in reactant flux after the catalyst particle, which cannot be described by reaction on the particle and diffusion alone. It is therefore relevant to discuss possible reasons for this behavior. The first one is photo bleaching during readout. However, control measurements at different excitation light intensities and exposure times show that this effect is negligible (Supplementary Fig. 15) and, furthermore, any photobleaching still occurring is accounted for by normalization using the fluorescence intensity signal from the reference channels without Au catalyst particle (Supplementary Notes 4–6). The second possible reason is related to the fact that fluorescein binds to SiO_2_ surfaces^31^ and thus to the inner walls of the nanochannels. Accordingly, an analytical 1D model that assumes equilibrium between reactant concentration in solution and on the nanochannel walls, combined with diffusion of the molecules adsorbed to the channel walls (see Supplementary Note 7 for details), can indeed predict a fluorescence intensity distribution along the nanochannel that is in good agreement with the experimental observation, and thus suggests that such a process is taking place.

### Turnover frequency measurements on single nanoparticles

**Fig. 3:**
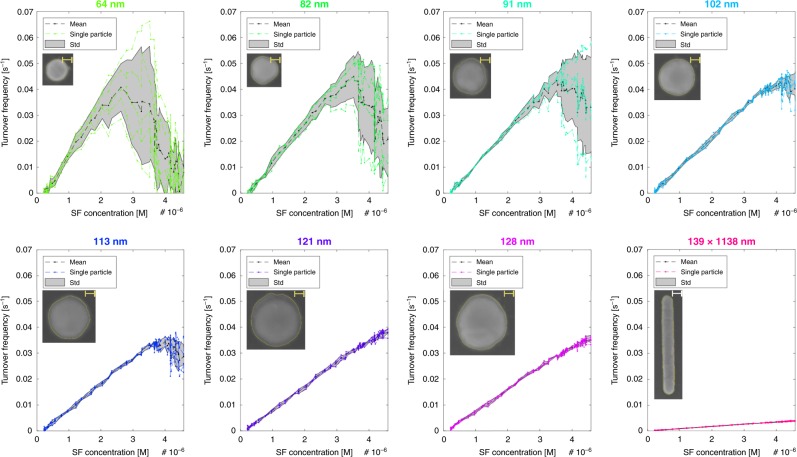
Summary of single particle-specific and mean turnover frequencies (ToFs) for 32 nanoparticles. All particles were addressed simultaneously in a single experiment at different nominal incoming fluorescein concentrations. The titles display the nominal particle diameters and the insets show SEM images of corresponding particle analogs taken after the cleaning step on an open surface. The scale bars are 20 nm in all insets except for the 139 × 1138 nm patch where it is 100 nm. Black and colored lines display mean and individual particle ToFs, respectively, and the gray shaded areas depict the standard deviation. The fluorescein concentration was systematically varied between 0 and 4.6 µM and the borohydride concentration was kept constant at 50 mM

Now turning back to single-particle activity, by measuring the flow rate in the nanochannels (Supplementary Figs. 16–18) and by estimating the number of surface atoms of the nanoparticles (Supplementary Fig. 4), we were able to estimate the ToFs of each individual nanoparticle over a wide range of reaction conditions (Supplementary Fig. 12a). The correspondingly obtained ToFs for the reduction of fluorescein by borohydride (50 mM), measured simultaneously for 32 single Au nanoparticles (three channels were clogged) as a function of fluorescein concentration in the reactant flow, are summarized in Fig. 3 and Supplementary Figs. 19–21. We make the following key observations. Firstly, at low fluorescein concentration, the ToF is a linear function of fluorescein concentration and does not show any sign of particle-specific response. We interpret this as the reaction being entirely mass transport limited, i.e. that flow and diffusion of fluorescein to the nanoparticle surface determines the reaction rate. We also note that the slope decreases with increasing particle size since ToF scales inversely with the amount of surface atoms in the mass transport limited regime according to the relation:

1 $${\mathrm{ToF}} = \frac{{C \cdot V}}{N},$$

where *C* is fluorescein concentration, *V* is the volume flowing past a particle per second, and *N* is the number of catalytic sites on the particle (Supplementary Fig. 22). Secondly, as we further increase the fluorescein concentration, a spread in ToF between individual particles for each set of size starts to appear. We observe a maximal ToF of 0.065 s^−1^, and a minimal ToF of 0.025 s^−1^ at identical conditions for two different nanoparticles in the set of the smallest nominal particle size of 64 nm (Fig. 3). Similarly, we find the maximum ToF at different fluorescein concentrations, which, as a general trend, increases with particle size. At the same time, both the maximal ToF and the observed particle-particle spread decreases with increasing particle size, and disappears completely for the 139 × 1138 nm Au patch. This can be understood as that for the largest nanoparticles we never reach the surface reaction limited regime and that the reaction is mostly (or completely in the case of the patches) mass transport limited. In other words, our system enables a systematic visualization of the interplay between mass transport and surface reaction control imposed by nanoconfinement, as for instance is characteristic for meso- or nanoporous support materials used in industrial catalysts.

Focusing on the smaller particle size regime only (64–91 nm, Fig. 3), we notice that the ToF starts to decrease for the highest considered fluorescein concentrations. This is in line with the Langmuir-Hinshelwood mechanism^32^, which states that two reactants first adsorb on the catalyst before the reaction takes place. The reaction rate is then governed by the interplay between both reactant concentrations and adsorption and desorption constants, which means that when the surface concentration of fluorescein becomes larger than the surface concentration of borohydride the ToF will decrease due to poisoning of the Au surface by fluorescein. However, the ToF dependence found for our system shows a steeper decrease at higher fluorescein concentrations than predicted purely by a Langmuir–Hinshelwood mechanism (Supplementary Fig. 23). This could either indicate that the reaction is not of the Langmuir–Hinshelwood type or that the reaction step is not rate determining, which would render the approximation invalid. Regardless, it is most certainly mainly a consequence of the tiny volume of the nanochannel (8.75 µm^3^ for the entire channel), which accelerates the transition between the mass transport and the surface reaction limited regimes, since when most of the molecules passing the particle also react, the limited mass transport will lead to a lower local reactant concentration in solution. This drop in local concentration in turn causes the observed steeper and more immediate decrease in activity observed at the highest fluorescein concentrations for all our experiments (Fig. 3 and Supplementary Figs. 19–21).

**Fig. 4:**
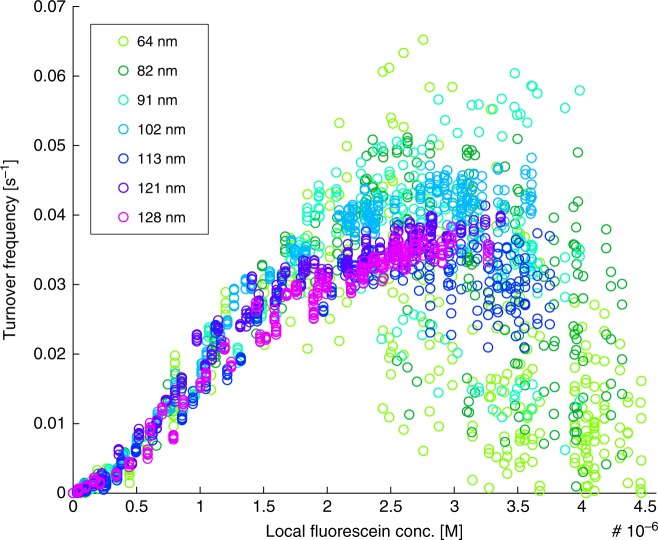
ToFs as function of local fluorescein concentration. The ToFs were derived from the measured absolute fluorescence intensity at the particle position. Note that ToFs for differently sized particles overlap in the mass transport limited regime but diverge in the surface reaction limited regime (local concentrations higher than ~2 µM)

As the next analysis step, we evaluate particle-specific ToFs as a function of the local fluorescein concentration measured at the position of each nanoparticle, based on the evolution of the fluorescence intensity distribution along the nanochannel (compare Fig. 2a and Supplementary Fig. 11) at each given time during the experiment. In other words, we derive the local fluorescein concentration from the measured fluorescence intensity at the position of each nanoparticle since it is this concentration (rather than the nominal concentration) that dictates the reaction rate. When plotting our data in this way, the activity at low local fluorescein concentrations becomes nearly equal for all particle sizes (Fig. 4). This corroborates our interpretation that in this regime the reaction is mass transport limited for all particle sizes and that site-specific activity is not important. It is also in good agreement with the calculated reaction rates in this regime, assuming a completely mass transport limited system (Eq. 1, Fig. 4, Supplementary Figs. 22 and 23). It is then not until local fluorescein concentrations higher than ~2 µM that we start to transit to the surface reaction limited regime where the ToFs become more and more particle specific, again with the largest spread in particle-specific activity for the smallest particle size considered.

### Trends in particle-specific activity

**Fig. 5:**
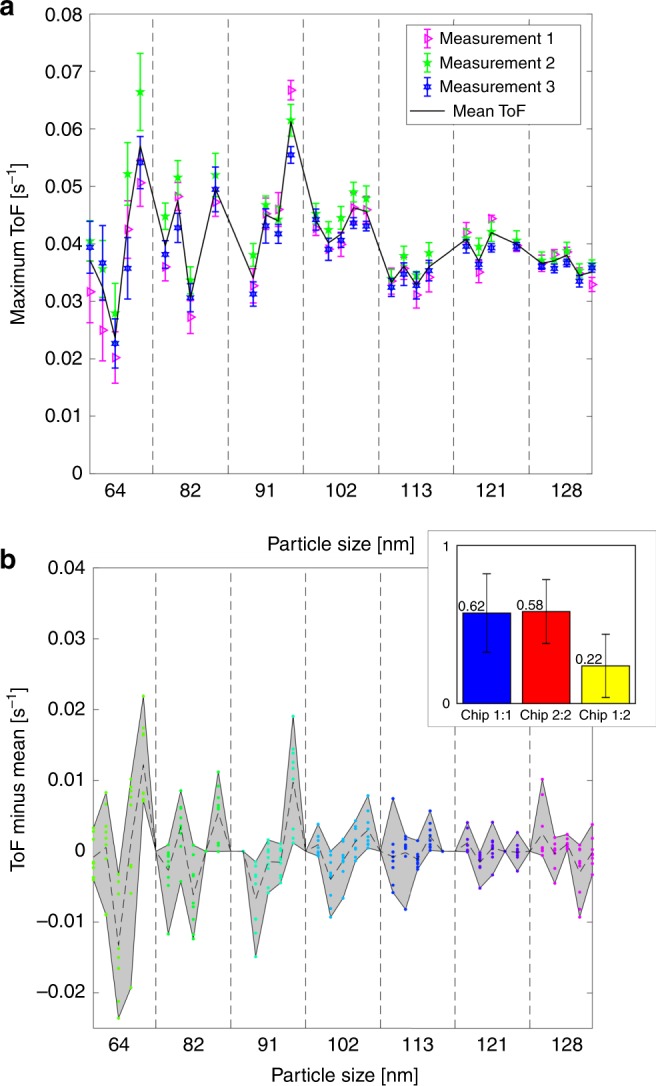
Particle-specific ToFs obtained in multiple experiments. **a** Three separate measurements executed with an initial incoming fluorescein concentration of 4.6 µM and constant sodium borohydride concentration of 50 mM. The error bars display the standard deviation for 10 values of the ToF measured at the nominal fluorescein concentration at the beginning of the experiment. Note that the same particles exhibit the highest/lowest activities in all experiments. **b** ToF with the subtracted mean value for each particle obtained from a total of nine independent experiments (such as the ones displayed in Fig. 3 and Supplementary Figs. 19–21) carried out on the same sample (chip 1). The colored dots (of same color code as in the previous figures) indicate the specific values of ToF with subtracted mean obtained for each particle in each measurement, the gray area depicts the span between the highest and lowest values and the dotted line corresponds to the mean value of each particle for all nine measurements. The inset displays the correlation coefficients between all ToFs with subtracted mean values for all measurements on two different but nominally identical samples, chip 1 & chip 2. In blue: only measurements from chip 1, in red: only measurements from chip 2, and in yellow: measurements in chip 1 correlated with measurements in chip 2. Error bars in the inset display standard deviation

Having discussed the overall evolution of measured ToFs as a function of reactant concentration, it is interesting to further analyze the surface reaction limited regime in detail because there the single particle-specific response is most apparent and our data show that some nanoparticles are more active than others. This is exemplified in Fig. 5a, in which the maximum ToF is shown for each particle on the chip in three independent experiments (note that the maximal ToF values displayed were obtained at different nominal fluorescein concentrations for the different particle sizes). Clearly, on average, catalyst activity appears to be independent of particle size, while at the same time the spread within the population increases as particle size is decreased (Fig. 5a and Supplementary Fig. 24). To understand these trends, we remind ourselves that all considered particles are in a size regime (64–128 nm) where no traditional scalable size effects are expected. Hence, the observed spread in activity for a certain size can be assigned to the different abundance of low coordination and defect sites imposed by the microstructure^5,29,33–35^. This becomes apparent from the TEM images of a large set of representative particles that exhibit widely varying structure and abundance of highly defectuous grain boundaries, containing copious low coordination sites due to lattice orientation mismatch between crystallites (Supplementary Figs. 2, 25)^33,36^. The observed decreased spread in activity between particles for increasing size can also be attributed to a larger degree of mass transport limitation for larger particles due to the restricted reactant supply by the nanochannel. This restriction is evident for sizes 102–128 nm in Fig. 3, but is not found across all experiments when different reaction conditions are applied (Supplementary Figs. 19–21).

To even further analyze and corroborate the single particle-specific activity, we resort to a larger data set and include nine independent measurements made on two nominally identical samples (chip 1 and chip 2—Fig. 5b and Supplementary Figs. 26 and 27). The particles retain their specific response also across a multitude of measurements, as specifically highlighted by subtracting the mean ToF from the 9 independent experiments from each data point. Furthermore, to also address this in a mathematically robust way: we calculated the correlation coefficients ($\frac{{{\mathrm{Cov}}(f,g)}}{{\sigma _f \cdot \sigma _g}}$, where *f* and *g* are measurement series values and *σ*_*i*_ are their standard deviation) between ToFs with subtracted mean for both chips (inset Fig. 5b and Supplementary Fig. 28). For each combination of experiments executed within one chip, we find correlation coefficients of 0.62 and 0.58 (inset in Fig. 5b and Supplementary Fig. 28). The much lower correlation coefficient of 0.22 between measurements in the two different chips thus indicates that the correlations for individual particle response between measurements is not an artefact imposed by how the measurements are performed, but a true effect related to the particles themselves.

## Discussion

We have presented a nanofluidic device that enables simultaneous and parallel scrutiny of catalyst activity in the liquid phase on several tens of individual catalyst nanoparticles at practically relevant (µM) reactant concentrations. In our platform, each catalyst nanoparticle is located in its own nanofluidic channel, which enables laminar flow of reactants to, and products from, the particles. As a second key feature, the nanofluidic design, with each nanoparticle isolated inside its own nanochannel, ensures that the reaction conditions are identical for all the catalyst particles, and that up- or downstream conversion effects by neighboring particles can be eliminated. Thirdly, the nanochannels retain the fluorescent molecules within the focal plane of the microscope during the entire experiment and thus enable the parallel scrutiny of individual catalyst nanoparticles under a wide range of reaction conditions. Specifically, as we have demonstrated here, the transition between the mass transport and surface reaction limited regimes can be visualized at the single nanoparticle level and analyzed as a function of nanoparticle size. Using the catalytic reduction of fluorescein by borohydride on Au nanoparticles as the model reaction, we found that in the mass transport limited regime all nanoparticles ranging in diameter from 60 to 130 nm exhibit essentially identical ToFs increasing linearly from 0 to ca. 0.01–0.03 s^−1^ per site, for local reactant concentrations increasing from 0 to 4.6 µM. In contrast, in the surface reaction limited regime, we observed distinct single particle-specific activity with some nanoparticles consistently being more active than others, as statistically verified over nine different experiments using the same nanofluidic chip. These effects could be explained by the different relative abundance of low coordination and defect sites due to a widely varying grain-microstructure between the nanoparticles, identified by ex situ TEM. Furthermore, the also observed decreased spread in activity between particles for increasing nanoparticle size could be attributed to a larger degree of mass transport control for larger particles, due to the limited reactant supply by the tiny nanochannel. In comparison with the state-of-the-art single-particle catalysis methods of SMFM (which give insights in the spatial position of the active sites due to superior spatial resolution) and SERS (which offers specific chemical information), our platform enables catalyst operation at technically relevant reactant concentrations and resolves reactant concentration gradients up- and downstream of single catalyst particles. Looking forward, we predict that our platform will not only be usable in combination with traditional fluorescence microscopy readout but also with super-resolution microscopy techniques and with micro-Raman spectroscopy to enable deeper chemical insights. Furthermore, either by utilizing nanofabrication for particles in the sub-10 nm size range^37,38^ or by specifically placing individual colloidal nanocrystals inside nanofluidic channels, it will become possible to extend the approach down into a size or structure range directly relevant for real catalysis beyond model systems, and to other types of reactions where a fluorescent product is formed, as demonstrated in a preliminary fashion in Supplementary Note 18 (Supplementary Figs. 29 and 30) for the catalytic oxidation of amplex ultra red to resorufin over an Au nanoparticle catalyst. Furthermore, in principle, our nanofluidic reactor platform can be applied to SMFM, SERS, and/or SPS readout to achieve a wider measurable concentration range and in this way compensate for some of the drawbacks of these techniques. Hence this platform will boost the field of single nanoparticle catalysis by enabling highly parallelized scrutiny of individual nanoparticles under perfectly controlled reaction conditions and at practically relevant reactant concentrations in solution, and by facilitating systematic visualization of the interplay between mass transport and surface reaction control imposed by nanoconfinement typical for meso- or nanoporous support materials used in industrial catalysts.

## Methods

### Nanofluidic chip fabrication

Fabrication of the nanofluidic chips was carried out in cleanroom facilities of Fed. Std.209 E Class 10–100, using electron-beam lithography (JBX-9300FS/JEOL Ltd), photolithography (MA 6/Suss MicroTec), reactive-ion etching (Plasmalab 100 ICP180/Oxford Plasma Technology and STS ICP), electron-beam evaporation (PVD 225/Lesker), magnetron sputtering (MS150/FHR), deep reactive-ion etching (STS ICP/STS) and wet oxidation (wet oxidation/Centrotherm), fusion bonding (AWF 12/65/Lenton), and dicing (DAD3350/Disco). In particular, the fabrication comprised several processing steps (Supplementary Note 1) of a 4”-silicon (p-type) wafer.

### Fluorescein reduction by borohydride on Au nanoparticles

Reagents (Fluorescein sodium salt, BioReagent, suitable for fluorescence, 46960, Sodium borohydride, powder, ≥98.0 %, 452882 and Au nanoparticles, 100 nm diameter, OD 1, stabilized suspension in citrate buffer, 742031) were purchased from Sigma Aldrich. Fresh stock solution of 2 mM fluorescein was prepared daily by dissolving 7.52 mg fluorescein salt in 10 ml of milli-Q water. Freshly prepared 100 mM aqueous solution of borohydride (37.83 mg in 10 ml) was used for each experiment due to the unstable nature of borohydride in water^39^. The stock solutions were then diluted and mixed to obtain a reaction mixture containing concentrations of fluorescein varying from 2.3 to 7.8 µM and 50 mM borohydride. Further, the reaction mixtures were directly injected into one of the inlets of the nanofluidic chip. The fluorescein concentration was confirmed by absorption measurements using a nanodrop 1000 spectrophotometer.

Before starting each experiment, the nanofluidic chip was flushed for 20 min with a solution of 4.3 wt% ammonia and 4.3 wt% hydrogen peroxide in milli-Q water to clean the nanoparticle surfaces. The chip was then flushed with milli-Q water twice to remove residues of the cleaning reagents. Measurements were then performed by flowing the reaction solution through an array of nanochannels, each containing a single Au nanoparticle, and evaluating the fluorescence signal after each particle. The evaluation was performed using in-house Matlab scripts to calculate ToFs for each channel/particle. The flow rate was during measurements kept constant at 145 µm per s (Supplementary Fig. 17).

### Characterization of Au nanoparticles

The microstructure of the used Au nanoparticles was characterized using a TEM, FEI Tecnai T20 operated at the accelerating voltage 200 kV. Nanoparticle sizes were determined using an SEM, Zeiss Supra 55—EDX operated at the accelerating voltage 15 kV. Plasmonic spectra of the Au nanoparticles were measured with dark-field scattering, using a Nikon eclipse LV150N microscope with a Nikon TU plan ELWD 50x NA = 0.6 objective, a 50 W halogen lamp, an Andor Shamrock SR-193i spectrometer with grating blaze 800 nm and a grating density of 150 g per mm and an Andor Newton 920 CCD camera. Additional details are found in Supplementary Note 2.

### Synthesis, purification and characterization of reduced fluorescein

An aqueous solution of fluorescein (50 mM) was stirred at room temperature with sodium borohydride (1 M) and Au nanoparticles (100 nm, 9.5 × 10^7^ particles per ml) for 3 h, protected from light, to completely reduce the fluorescein (Supplementary Fig. 7). The reaction mixture was then acidified to pH 4 by addition of 10% citric acid solution and extracted three times with 10 mL ethyl acetate. Organic solvent, as well as citric acid solution, was degassed with nitrogen prior the work up of the product to suppress aerobic oxidation. The organic aliquots were combined, dried over anhydrous sodium sulphate and concentrated in vacuo. The residue was dried under high vacuum for 24 hours to yield the air sensitive product as a yellow film. The product residue was characterized with NMR, HRMS, and IR (Supplementary Note 3).

### Diffusion and reaction simulations

1D and 3D simulations of diffusion and the reaction in a nanochannel were performed using in-house Matlab scripts (1D) and computational fluid dynamics (CFD) code ANSYS Fluent 15.0.7 (3D). Details are found in Supplementary Notes 7, 8, and 10.

## Supplementary information

Supplementary Information

Peer Review

## Data Availability

The data that support the findings of this study are available from the corresponding authors upon request.
