# Supplementary material for: A nanofluidic device for parallel single nanoparticle catalysis in solution
Source: Nat Commun. 2019 Sep 27;10:4426. doi: 10.1038/s41467-019-12458-1 (PMC6764984; doi:10.1038/s41467-019-12458-1)
Supplement: Supplementary file 1 — Supplementary Information [file 41467_2019_12458_MOESM1_ESM.pdf]

Supplementary information

A nanofluidic device for parallel single nanoparticle catalysis in solution

Levin et al.

Contents

Supplementary information 1
Supplementary Methods 3
Supplementary Discussion 23
Supplementary References 32

Supplementary Methods

All fluorescence microscopy experiments were performed using a Zeiss Axio Observer Z1 microscope, with a Colibri 7 LED light source (wavelength 475 nm), an Andor iXon Ultra 888 EMCCD camera and an alpha Plan-Apochromat 63x/1.46 Oil Corr M27 objective. Images were taken with 100 ms exposure time and delay of 10 seconds between images to minimize effects of photobleaching. The fabrication comprised the following processing steps of a 4"-silicon (p-type) wafer:

Supplementary Note 1

Nanofluidic chip fabrication

Thermal oxidation: (a) Cleaning for 10 min at 80˚C in 1:1:5 H_2O_2:NH_3OH:H_2O (SC-1), rinsing in water, HF-dip for 30 sec, cleaning for 10 min at 80˚C in 1:1:5 H_2O_2:HCl:H_2O (SC-2), rinse in water, and drying under N_2-stream. (b) Wet oxidation in water atmosphere for 660 min at 1050˚C (2000 nm thermal oxide).

Fabrication of alignment marks: (a) Spin coating HMDS adhesion promoter (MicroChem) at 3000 rpm for 30 sec and soft baking on a hotplate (HP) at 115˚C for 120 sec. Spin coating UV5 (MicroChem) at 3000 rpm for 60 sec and soft baking (HP) at 130˚C for 120 sec. (b) Electron-beam exposure of alignment marks for both optical and electron-beam lithography at 10 nA with a shot pitch of 24 nm and 25 µCcm^{-2} exposure dose. (c) Post-exposure bake (HP) at 130˚C for 90 sec. (d) Development in MF-24A (Microposit) for 90 sec, rinsing in water and drying under N_2-stream. (e) Reactive-ion etching (RIE) for 15 sec at 60 mTorr chamber pressure, 60 W RF-power, 60 sccm O_2-flow (descum). RIE for 20 min at 30 mTorr chamber pressure, 150 W RF-power, 50 sccm Ar-flow, 50 sccm CHF_3-flow, (800 nm etch depth in thermal oxide). (f) Removal of resist-mask in 50 mL H_2O_2 + 100 mL H_2SO_4 at 130˚C for 10 min, rinsing in water, and drying under N_2-stream.

Fabrication of nanochannels: (a) Electron-beam evaporation of 20 nm Cr (hard mask). (b) Spin coating ZEP520A:anisole (2:1) (ZEONREX Electronic Chemicals) at 2000 rpm for 60 sec and soft baking (HP) at 180˚C for 10 min. (c) Electron-beam exposure of lines at 2 nA with a shot pitch of 4 nm and 280 µCcm^{-2} exposure dose. (d) Development in n-amyl acetate for 120 sec, rinsing in isopropanol and drying under N_2-stream. Covering alignment marks with Kapton tape. (e) RIE for 10 sec at 40 mTorr chamber pressure, 40 W RF-power, 40 sccm O2-flow (descum). RIE for 90 sec at 20 mTorr chamber pressure, 50 W RF-power, 200 W ICP-power, 20 sccm O_2-flow, 50 sccm Cl_2-flow (selective Cr hard-mask etch). RIE for 100 sec at 8 mTorr chamber pressure, 50 W RF-power, 50 sccm NF_3-flow (100 nm etch depth in thermal oxide). (f) Removal of Cr-mask in 50 mL H_2O_2 + 100 mL H_2SO_4 at 130˚C for 10 min, rinsing in water, wet-etching in standard Cr-wet etch, and drying under N_2-stream.

Fabrication of microchannels: (a) Spin coating HMDS at 3000 rpm for 30 sec and soft baking (HP) at 115˚C for 2 min. Spin coating S1813 (Shipley) at 3000 rpm for 30 sec and soft baking (HP) at 115˚C for 2 min. (b) Expose microchannels for 8 sec in contact aligner at 6 mWcm^{-2} intensity. (c) Development in MF-319 (Microposit) for 60 sec, rinsing in water and drying under N_2-stream. (d) Reactive-ion etching (RIE) for 15 sec at 60 mTorr chamber pressure, 60 W RF-power, 60 sccm O_2-flow (descum). RIE for 25 min at 30 mTorr chamber pressure, 150 W RF-power, 50 sccm Ar-flow, 50 sccm CHF_3-flow, (1000 nm etch depth in thermal oxide). (e) Removal of resist in 50 mL H_2O_2 + 100 mL H_2SO_4 at 130˚C for 10 min, rinsing in water and drying under N_2-stream.

Fabrication of inlets (from backside): (a) Magnetron-sputtering of 200 nm Al (hard mask). (b) Spin coating S1813 at 3000 rpm for 30 sec and soft baking (HP) at 115˚C for 2 min. (c) Expose inlets for 10 sec in contact aligner at 6 mWcm^{-2} intensity. (d) Development in MF-319 for 60 sec, rinsing in water and drying under N2-stream. (e) Aluminum wet etch (4:4:1:1 H_3PO_4:CH_3COOH:HNO_3:H_2O) for 10 min to clear the hard mask at inlet positions. (f) Deep reactive-ion etching for 300 cycles of 12 sec at 5 mTorr chamber pressure, 600 W RF-power, 10 W platen power, 130 sccm SF6-flow (Si-etch), and of 7 sec at 5 mTorr chamber pressure, 600 W RF-power, 10 W platen power, 85 sccm C_4F_8-flow (passivation) at a

rate of 2 µm per cycle. (g) Removal of Al-hard mask in 50 mL H_2O_2 + 100 mL H_2SO_4 at 130˚C for 10 min, rinsing in water and drying under N_2-stream.

Fabrication of nanoparticles inside nanochannels: (a) Spin coating Copolymer MMA(8.5)MMA (MicroChem Corporation, 10 wt % diluted in anisole) at 6000 rpm for 60 sec and soft baking (HP) at 180˚C for 10 min. Spin coating ZEP520A:anisole (1:2) at 3000 rpm for 60 sec and soft baking (HP) at 180˚C for 10 min. (b) Electron-beam exposure at 1 nA with a shot pitch of 2 nm and 280 μCcm^{-2} exposure dose. (c) Development in n-amyl acetate for 120 sec, rinsing in isopropanol and drying under N_2-stream. Development in methyl isobutyl ketone:isopropanol (1:1) for 120 sec, rinsing in isopropanol and drying under N_2-stream. (d) Electron-beam evaporation of 20 nm Au. (e) Lift-off in acetone, rinsing in isopropanol, and drying under N_2-stream.

Fusion bonding: (a) Cleaning of the substrate together with a lid (175 µm thick 4”-pyrex, UniversityWafers) in 5:1:1 H_2O:H_2O_2:NH_3OH (SC-1) for 10 min at 80˚C. (b) Pre-bonding the lid to the substrate by bringing surfaces together and manually applying pressure. (c) Fusion bonding of the lid to the substrate for 5 h in N_2 atmosphere at 550˚C (5˚C per min ramp rate).

Dicing of bonded wafers: Cutting nanofluidic chips from the bonded wafer using a resin bonded diamond blade of 250 µm thickness (Dicing Blade Technology) at 35 krpm and 1 mm per s feed rate.

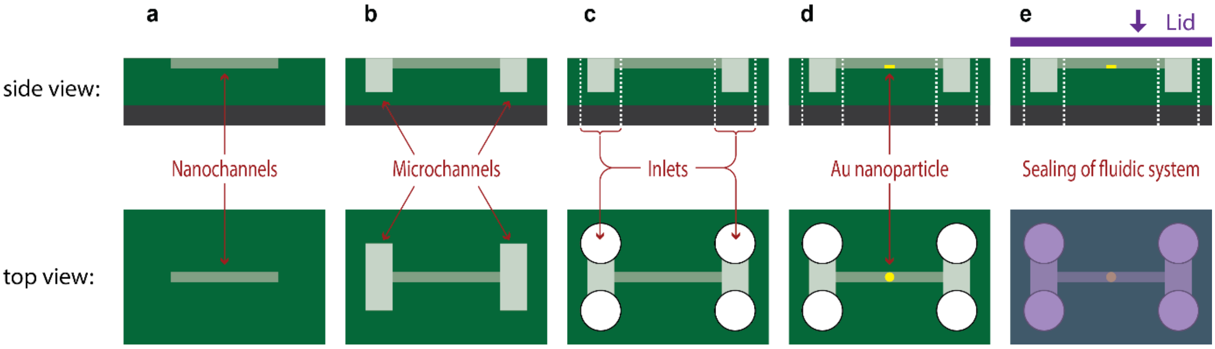

Supplementary Figure 1. Main processing steps for the fabrication of nanofluidic devices with catalytic nanoparticles, (a) reactive-ion etching of nanofluidic structures into the thermal oxide of a Si-substrate, (b) reactive-ion etching of a microfluidic structures into the thermal oxide of a Si-substrate, (c) deep reactive ion etching of inlets connecting to the fluidic structures, (d) fabrication of Au-nanoparticles inside the nanofluidic structures, and (e) sealing of the fluidic structures with a lid to form a closed fluidic system.

Supplementary Note 2

Characterization of Au nanoparticles

The microstructure of analogues to the used Au nanoparticles were characterized using a transmission electron microscope (FEI Tecnai T20 at accelerating voltage 200 kV) to gain insight into particle-to-particle heterogeneity (Supplementary Figure 2). For this purpose, as discussed in the main text, we used a sample analoge nanofabricated onto on a TEM membrane,[1] which was exposed to a thermal treatment mimicking the bonding step of the lid of the nanofluidic device to induce the same recrystallization effects. Since we the largest particle-to-particle differences in particle activity were found for small particles, we focused our analysis on a set of particle analoges (average diameter 65.8 ± 1 nm) similar in size to the smallest particles (64 nm) studied in the nanofluidic device. From the corresponding set of TEM micrograps of 22 nanoparticles, it becomes apparent that they display widely different microstructure in terms of grain boundaries, grain size and grain boundary type (Supplementary Figure 2), which is the likely cause of the variations in reactivity in our ToF measurements (Figure 5 and Supplementary Figure 25).

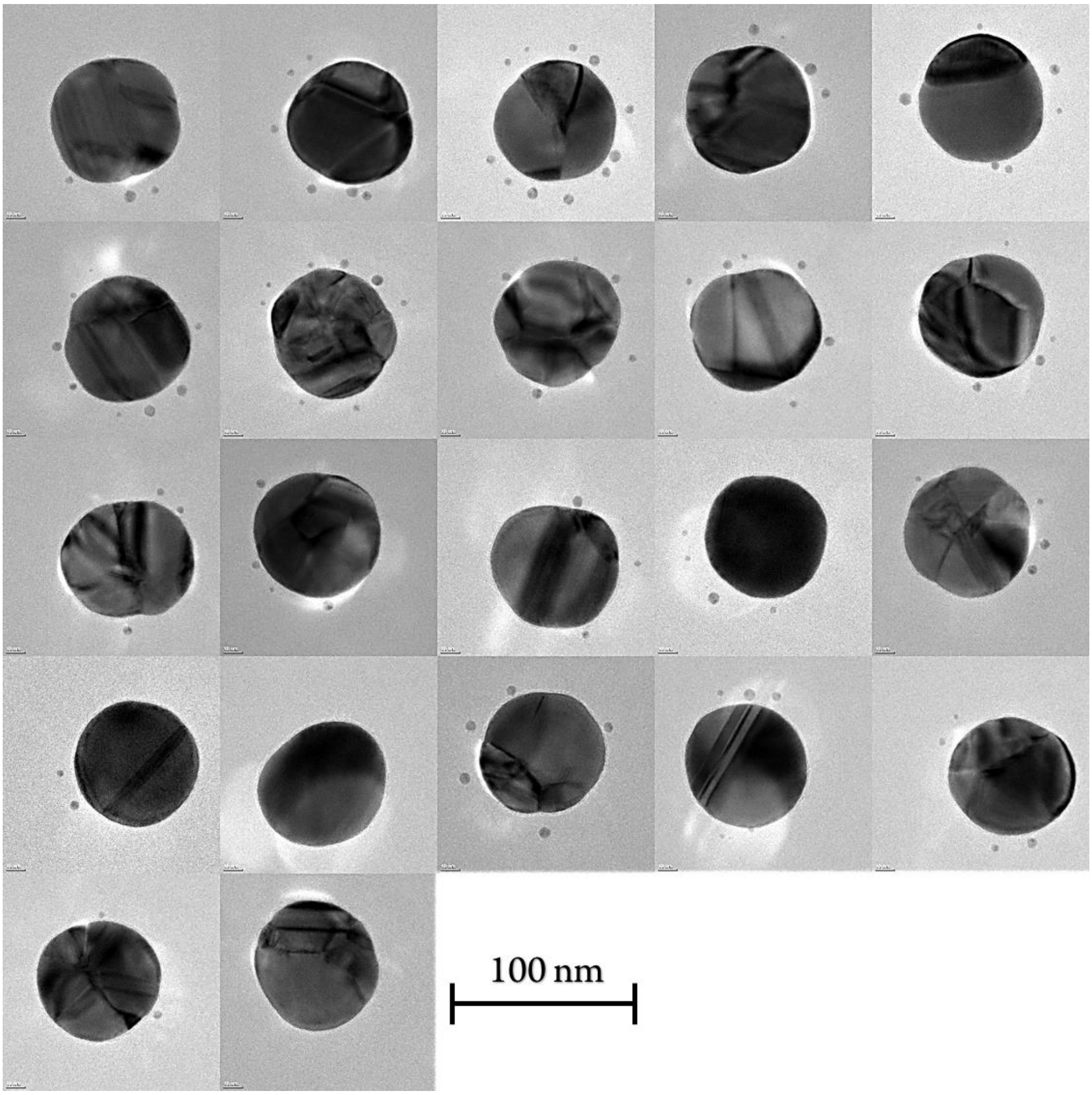

Supplementary Figure 2. TEM images of 22 Au nanoparticles of approximately 66 nm in diameter after thermal treatment mimicking the bonding step of the lid for during fabrication of the nanofluidic chip to induce the same type of recrystallization. The scale bar is 100 nm.

In Supplementary Figure 2, multiple smaller satellite particles, surrounding the larger nanoparticles, can also be seen. The satellites are, however, efficiently removed after several cleaning iterations (Supplementary Figure 3). Since the abundance of satellites around individual nanoparticles could cause different apparent activities, we have developed the cleaning procedure described above (solution of 4.3 wt% ammonia and 4.3 wt% hydrogen peroxide in milli-Q water) to remove these satellites prior to activity testing in the nanofluidic device. To demonstrate the effectiveness of this approach for getting rid of the satellites, we exposed the TEM sample multiple times by 10 minutes incubation. The result is summarized in Supplementary Figure 3, which shows that after each cleaning cycle the number of satellites is drastically reduced from on average ca. 20 satellites per particle to zero after three cleaning iterations. Since we have exposed our nanofluidics chips to at least two 20 min cleaning cycles prior to the experiments displayed in this work, no satellites are expected to be present in the nanofluidic device. To further examine the risk of Au binding to the sillica surface near the nanoparticles, Energy Dispersive X-ray Spectroscopy (EDS) was performed across the edge of a three times cleaned nanoparticle (Supplementary Figure 3c). No trace of Au was found surrounding the nanoparticle or its single satellite.

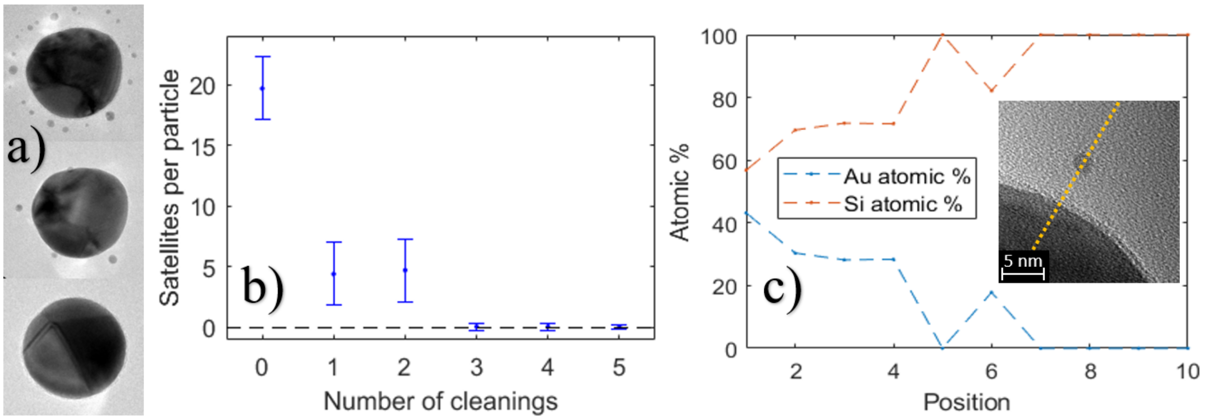

Supplementary Figure 3. a) TEM images of a nanoparticle before cleaning and after 2 and 5 cleaning cycles. The top and middle images show the same particle before and after cleaning twice. The bottom TEM image displays a typical nanoparticle after 5 cleaning cycles when all satellites have been removed. b) Average number of satellite particles counted for 22 Au nanoparticles of approximately 66 nm in size after an increasing number of cleaning cycles in solution of 4.3 wt% ammonia and 4.3 wt% hydrogen peroxide in milli-Q water. The error bars display standard deviation. c) TEM-EDS measurement displaying the amount of Au and Si across the edge of a nanoparticle. The distance between measurement positions was 4 nm and the inset shows the track along which the in total 10 positions were measured, starting from the large particle. Position 6 corresponds to the small satellite.

The structure of the Au nanoparticles was also characterized using scanning electron microscopy (SEM), both for particles located in nanochannels (prior to lid bonding) and on an open surface (Figure 1b and Supplementary Figure 4). The particles on the open surface were used to examine how the exposed particles change due to different treatments, such as annealing, cleaning and exposure to reactants (Supplementary Figure 4). Before annealing, the particles have cylindrical shape, exhibit a very rough microstructure with very small grains and with halos of small satellites around them (Supplementary Figure 4a). During annealing they recrystallize into mono- or polycrystals with few large grains and most small satellites coalesced into few larger ones (Supplementary Figure 4b). Subsequently, after cleaning in solution of 4.3 wt% ammonia and 4.3 wt% hydrogen peroxide in milli-Q water and exposure to reactants the overall structure is unchanged, except for the removal of the satellites discussed above (Supplementary Figure 4c-d; Supplementary Figure 3).

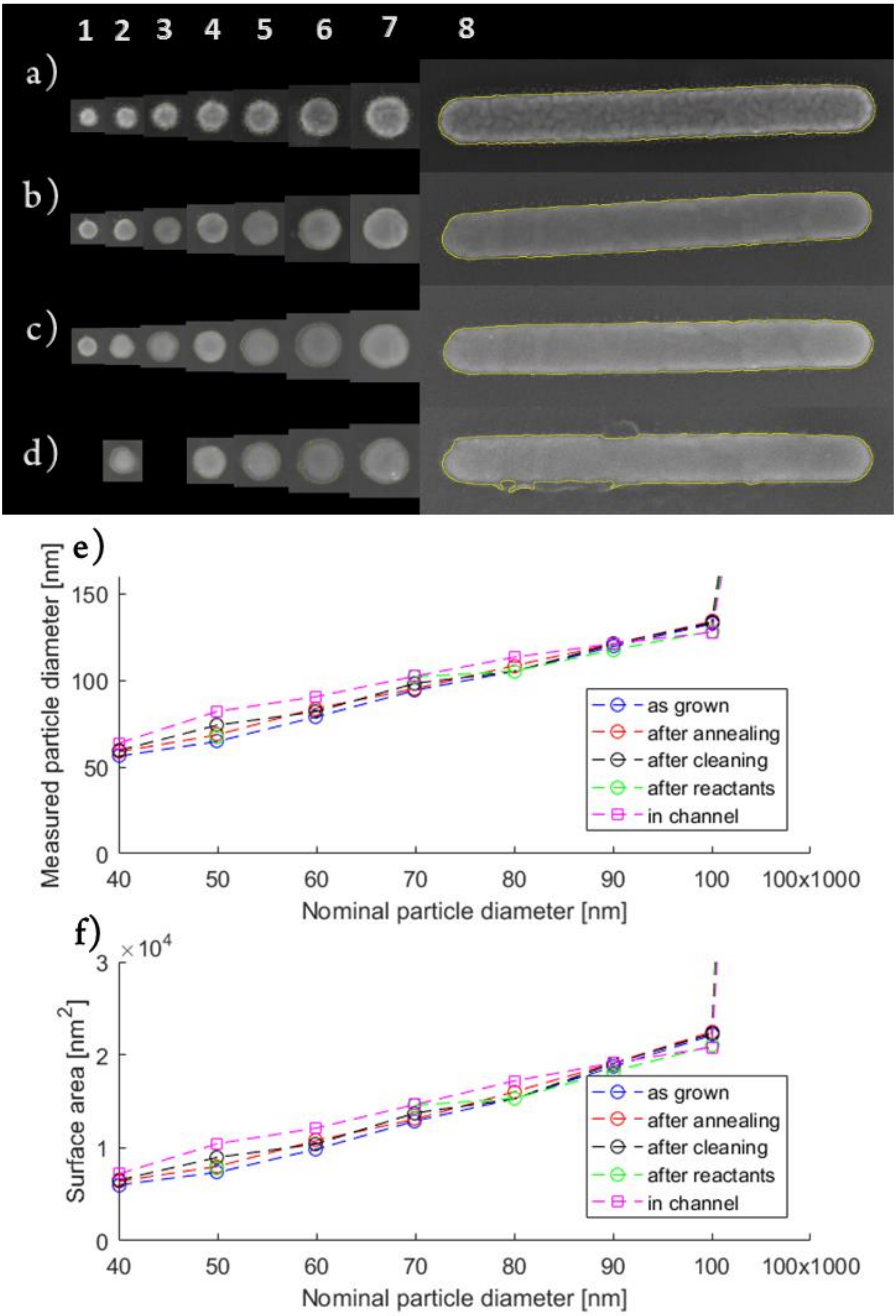

Supplementary Figure 4. SEM micrographs of Au nanoparticles of the same sizes as in the nanochannels but fabricated on an open surface to enable imaging and exposed to the same treatment as the particles in the nanochannels. All SEM images to the left show the differently sized particles a) before any treatment, b) after annealing, mimicking lid bonding, c) after cleaning in solution of 4.3 wt% ammonia and 4.3 wt% hydrogen peroxide in milli-Q water and d) after exposing to the reactants. The two particles that are missing could not be imaged due to contamination. e) Diameter before, after and in between every step of treatment. f) Estimated surface area derived from the particle diameters extracted from the images.

To be able to indirectly also characterize the Au particles located inside the nanochannel after lid bonding we employed plasmon resonance spectroscopy by measuring the dark-field scattering spectra of the individual Au nanoparticle inside the nanochannel[2] (in chip 2, Supplementary Figure 6). We found increasing scattering intensity with increasing particle size, as expected due to radiation damping and retardation effects[3], as well as quite significant variation among particles of the same nominal size, indicating minor variations in size, shape and microstructure, as discussed in the main text.

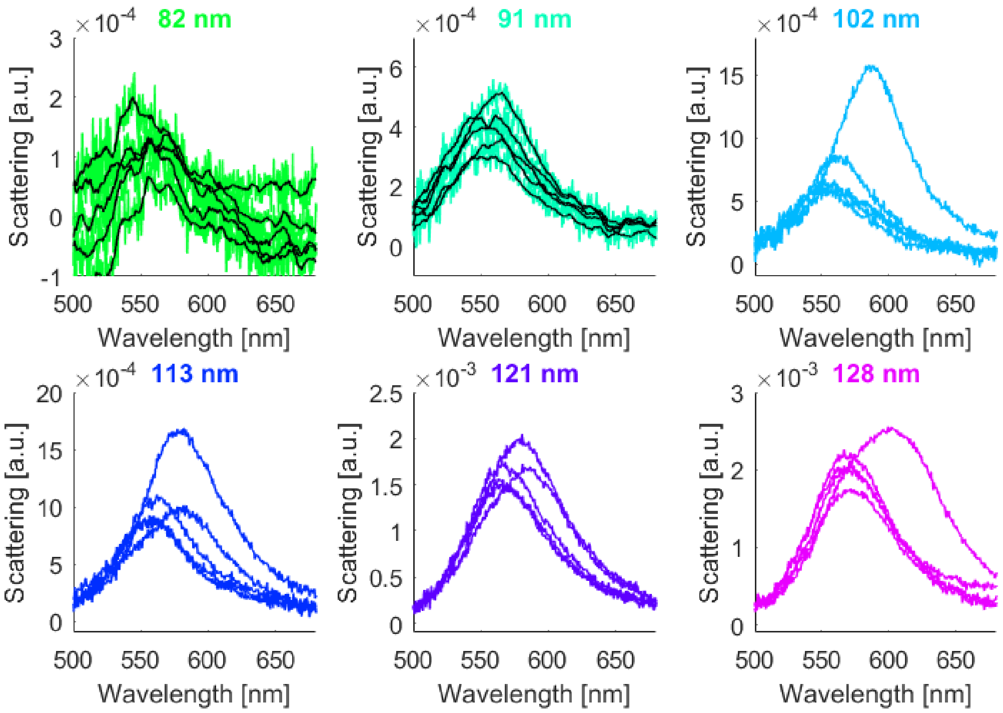

Supplementary Figure 5. Dark field scattering spectra of all Au nanoparticles located in the nanochannels of Chip 2 used in our experiments reported in the main text. They were recorded after cleaning by flushing aqueous solution of 4.3 wt% ammonia and 4.3 wt% hydrogen peroxide for 20 min. The smallest particle size on the chip (64 nm) was not measured because of a too small scattering cross section. Black lines for particles of size 82 and 91 nm are moving averages of 10 nearby values (corresponding to a width of 5 nm).

We also measured the single particle scattering spectra of selected nanoparticles for each size after cleaning and multiple reaction cycles (Supplementary Figure 6). Clearly, even after cleaning in aqueous solution of 4.3 wt% ammonia and 4.3 wt% hydrogen peroxide, the scattering spectra remained unchanged, corroborating that the cleaning procedure neither alters size/shape nor microstructure. However, after exposure to reactants, the scattering spectra appear red shifted, indicating bound molecules on the nanoparticle surface, as expected.

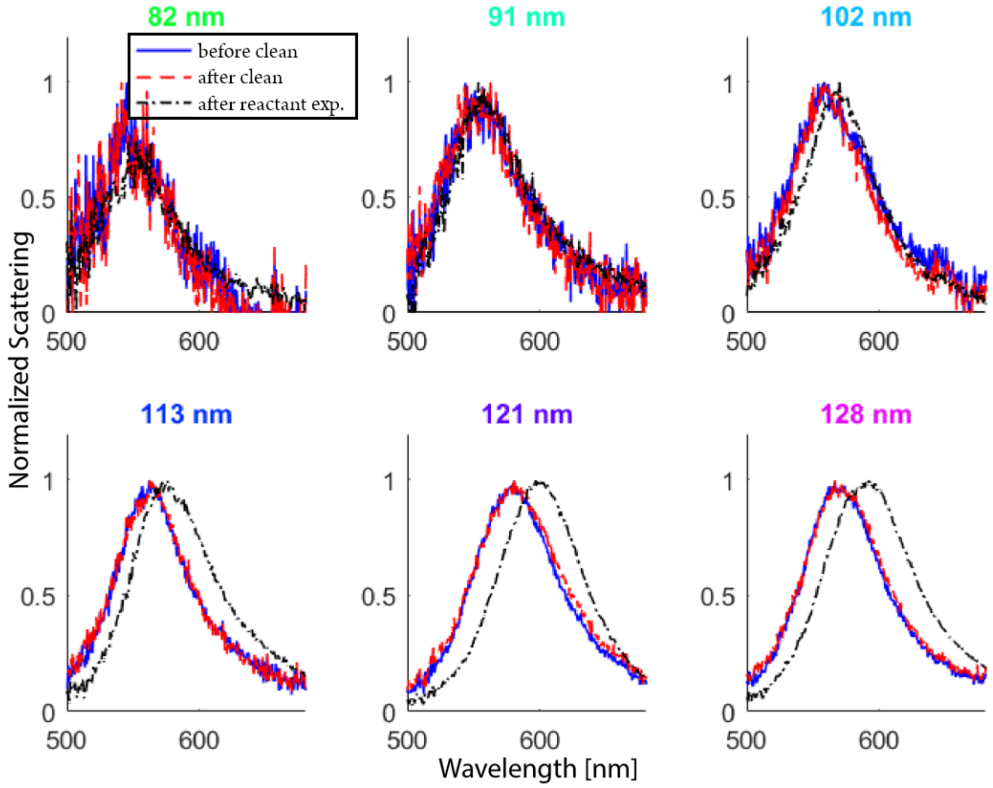

Supplementary Figure 6. Dark field scattering spectra of a selected Au nanoparticle for each size, measured inside a nanochannel before (blue lines) and after (red dashed lines) cleaning in aqueous solution of 4.3 wt% ammonia and 4.3 wt% hydrogen peroxide, as well as after exposure to reactants (black dashed lines). No noteworthy spectral changes can be observed after the cleaning, whereas a spectral redshift is observed after reaction, indicating a change in refractive index at the nanoparticle surface due to reactant adsorption.

Supplementary Note 3

Reaction product characterization

Reduction of fluorescein (Supplementary Figure 7) was performed and the product was characterized with various techniques. Nuclear magnetic resonance spectroscopy (NMR) spectra were obtained with a Varian 400. Chemical shifts are reported as part per million relative to the residual solvent peak from the solvent (CD_3OD): ^{1}H NMR at δ 3.31 ppm and ^{13}C NMR at δ 49.0 ppm. High-resolution mass spectrometry (HRMS) was performed at the Chalmers Mass Spectrometry Infrastructure using a HRMS Quadrupole Time-of-Flight (QTOF) Instrument (Agilent 1290 infinity LC system equipped with autoSampler tandem to an Agilent 6520 Accurate Mass Q-TOF LC/MS) with Agilent MassHunter Workstation software (LC/MS data acquisition for 6200 series tof/6500 series q-tof version b.06.01). Infrared spectroscopy (IR) was carried out using a PerkinElmer Frontier FT-IR.

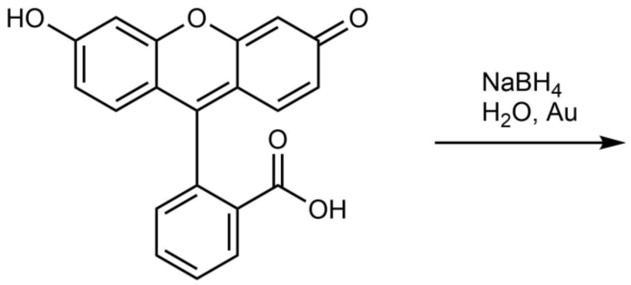

Supplementary Figure 7. The reaction scheme for the reduction of fluorescein.

The results from various characterization techniques agree that the reaction of fluorescein and borohydride on Au nanoparticles result in the reduced fluorescein displayed in Supplementary Figure 7: ^{1}H NMR (400 MHz, Methanol-d_4) δ 4.93 (s, 2H, Ar-O<u>H</u>), 6.22 (s, 1H, Ar_3-<u>H</u>), 6.40 (ddd, J = 8.4, 2.5, 0.8 Hz, 2H, Ar-<u>H</u>), 6.51 (dd, J = 2.5, 0.8 Hz, 2H, Ar-<u>H</u>), 6.86 (dd, J = 8.4, 0.8 Hz, 2H, Ar-<u>H</u>), 6.96 – 7.01 (m, 1H, Ar-<u>H</u>), 7.16 (td, J = 7.5, 1.3 Hz, 1H, Ar-<u>H</u>), 7.27 (td, J = 7.6, 1.5 Hz, 1H, Ar-<u>H</u>), 7.75 – 7.81 (m, 1H, Ar-<u>H</u>) (Supplementary Figure 8). ^{13}C NMR (101 MHz, Methanol-d_4) δ 172.1, 158.1, 152.8, 150.1, 132.9, 132.3, 131.6, 131.5, 130.3, 126.8, 117.4, 112.0, 103.6, 38.5 (Supplementary Figure 9). IR (ATR, ν_{max}/cm^{-1}): 3221, 2478, 1685, 1610, 1499, 1431, 1263, 1163, 1101, 991, 841, 747, 629, 459. HRMS (ESI-negative mode) *m/z* calculated for $C_{20}H_{13}O_5$ $[M-H]^-$: 333.0768; found 333.0780.

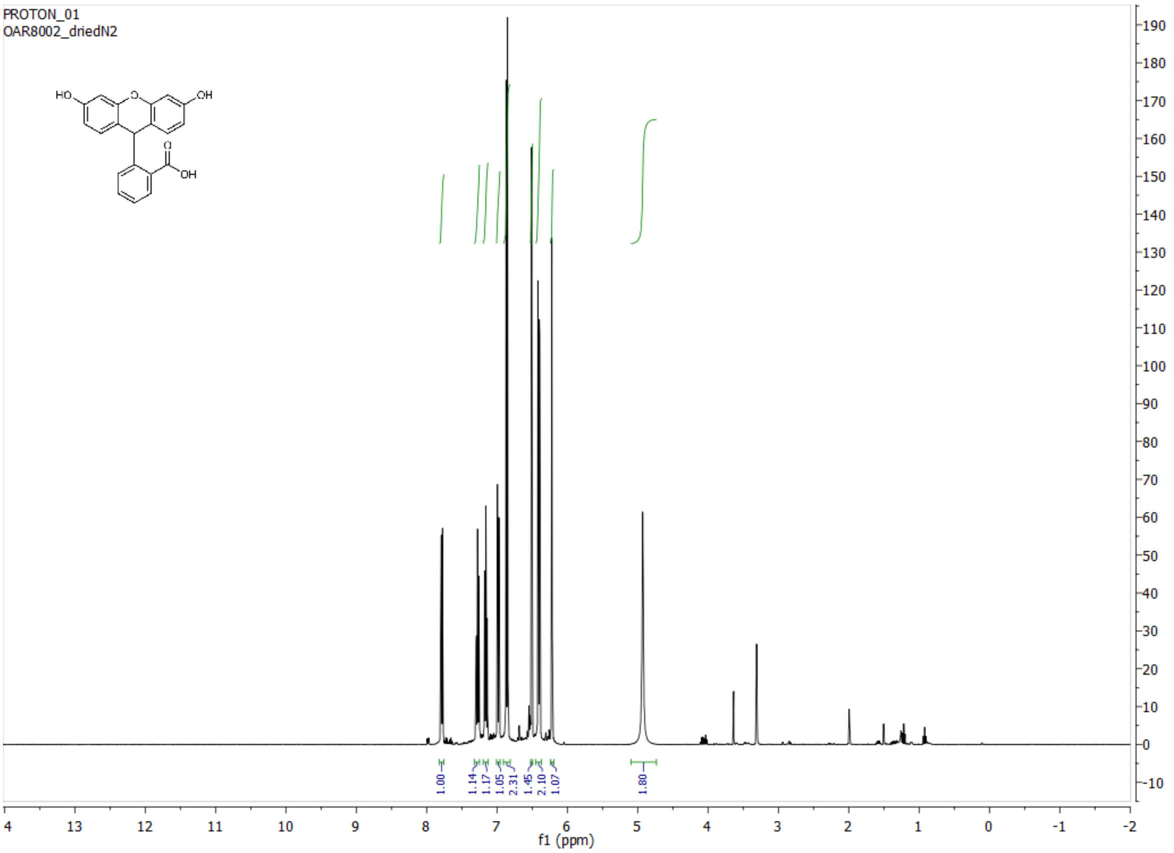

Supplementary Figure 8. ^{1}H NMR spectrum of the reduced fluorescein.

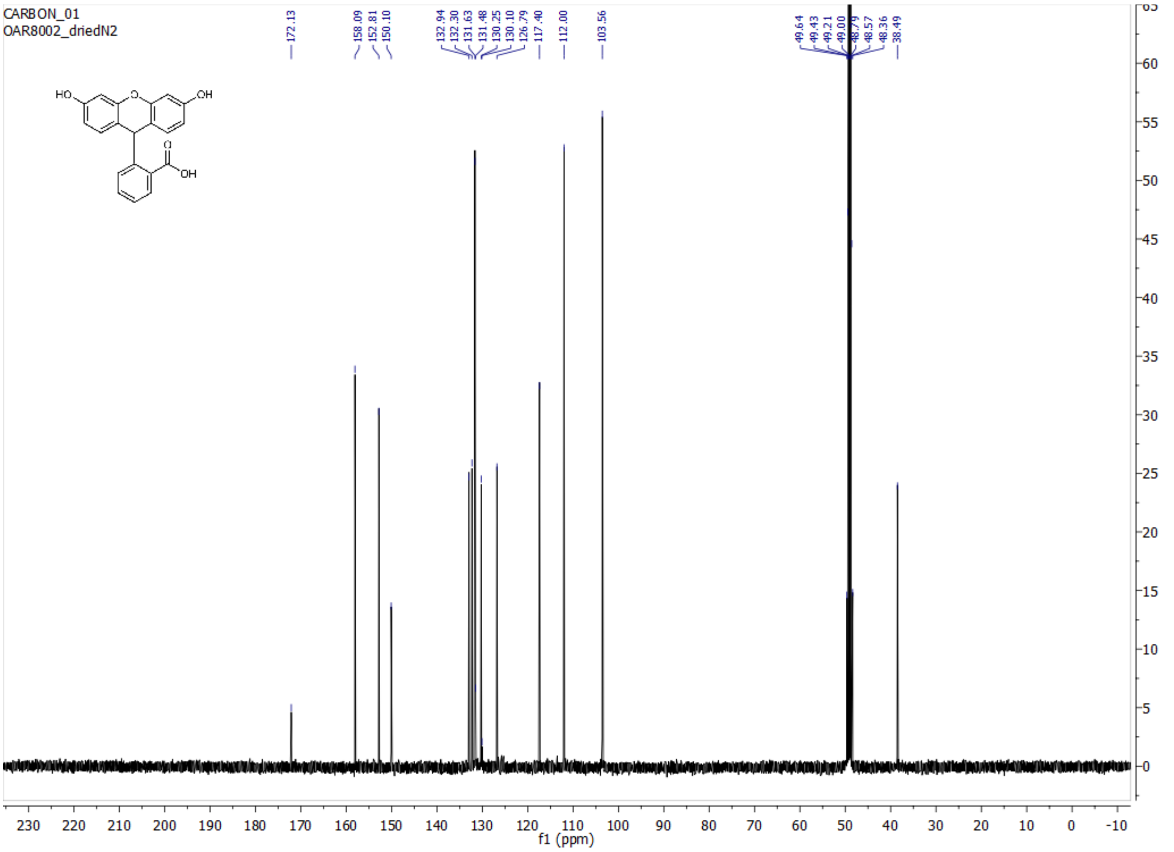

Supplementary Figure 9. ^{13}C NMR spectrum of the reduced fluorescein.

Supplementary Note 4

Measuring and interpreting raw data

Measurements of turnover frequencies (ToFs) were performed by flowing a reactant solution of 2.3-7.8 µM fluorescein and 50 mM borohydride in water at a constant flow rate of 145 µm per s through the array of nanochannels. We used two types of reference channels, which either contained a 139 x 1138 nm patch or no nanoparticle at all, to establish the two extreme situations when everything (I_1) or nothing (I_2) reacted, respectively. Based on these reference channels, the normalized intensity $I_{\mathrm{norm}} = \frac{I_{\mathrm{chan}}-I_1}{I_2-I_1}$ was calculated, where I_{chan} is the intensity of the evaluated channel, I_1 is the intensity in the reference channels where everything has reacted, and I_2 is the intensity in the reference channels without nanoparticle and thus without reaction (Supplementary Figure 10). I_{norm} then corresponds to the amount of fluorescein molecules that are left in the nanochannel after reaction on the catalyst.

The ToF of the reaction was then calculated according to:

$$\mathrm{ToF} = (1 - < I_{\mathrm{norm}} >_{\mathrm{roi}}) \cdot \frac{C \cdot V_{\mathrm{flow}}}{N_{\mathrm{sites}}} \qquad (1)$$

Where $< I_{\mathrm{norm}} >_{\mathrm{roi}}$ is the mean value of I_{norm} in the region of interest (Supplementary Figure 10), C is the incoming fluorescein concentration, V_{flow} is the volume flowing past a particle per second, and N_{sites} is the number of catalytic sites on the particle.

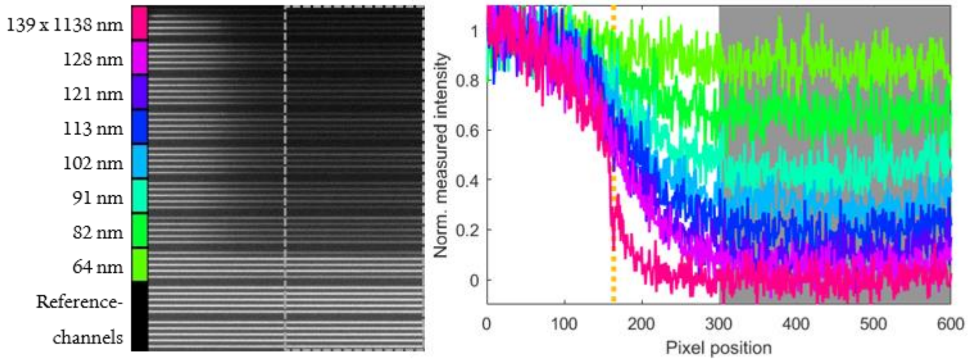

Supplementary Figure 10. The left image shows a typical CCD image of an experiment with color-coded nanochannel sets that contain Au particles of a certain size. The image to the right shows the normalized mean fluorescence intensity profile along each set of channels containing a certain particle size. The dashed gray area in the left image corresponds to the shaded gray area in the graph to the right and is the region of interest located downstream of the nanoparticles.

Supplementary Note 5

Fluorescein intensity distributions

The graphs in Supplementary Figure 11 show the mean (averaged over all 5 channels per particle size) fluorescence intensity profile along a channel for each particle size at different time points during an experiment, starting at high incoming reactant concentration (black lines 4.6 µM) and going to low incoming concentrations (colored lines - lowest 2.4 µM). The plotted fluorescence intensities are normalized with the corresponding intensity in the empty reference channels. The decreasing impact of change in incoming concentration for increasing particle size is again a manifestation of increasing mass transport limitations that control the reaction rate.

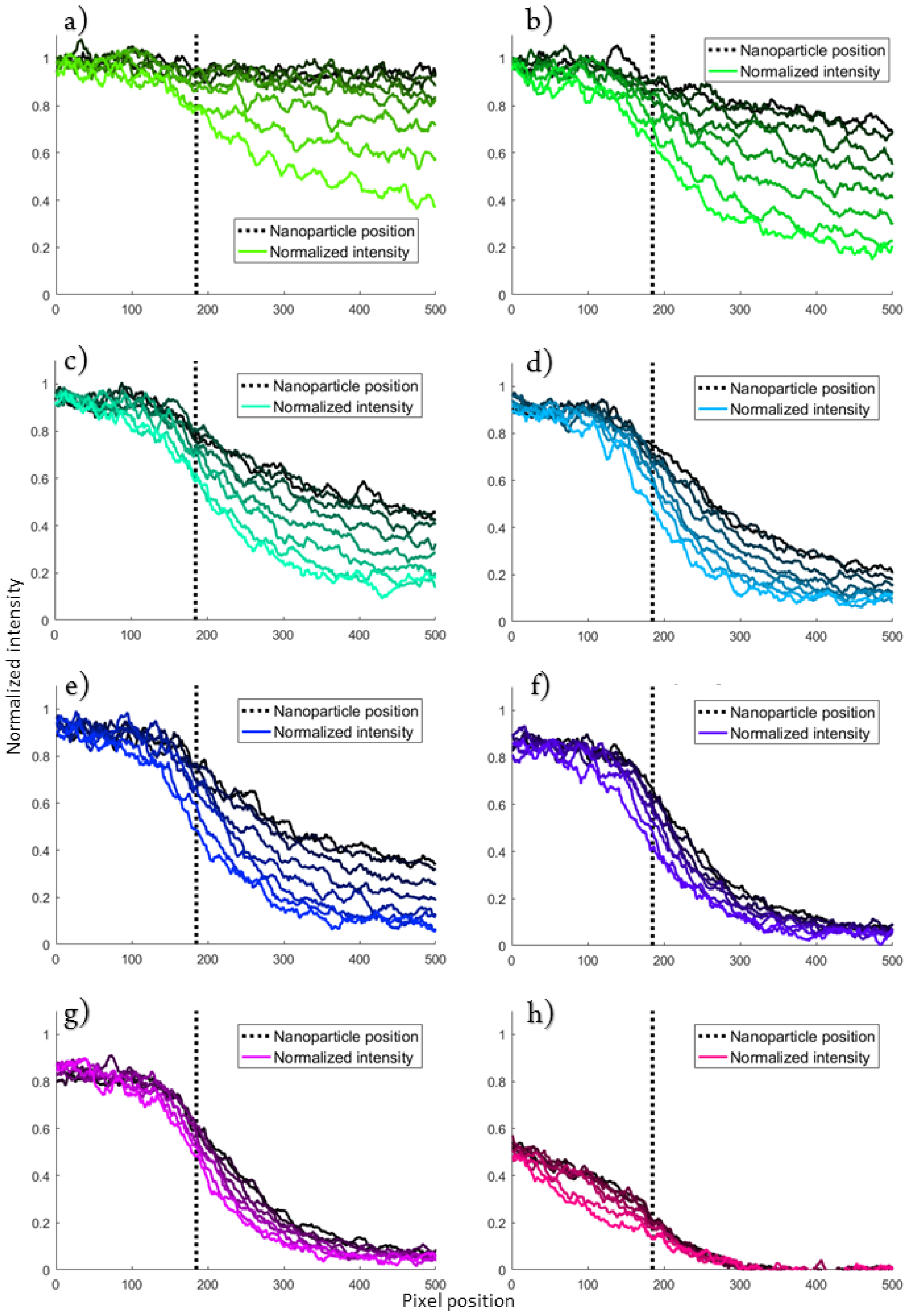

Supplementary Figure 11. Mean (over 5 particles) fluorescence intensity distribution evolution along the nanochannel for each nanoparticle size: a) 64 nm b) 82 nm c) 91 nm d) 102 nm e) 113 nm f) 121 nm g) 128 nm h) patch of 139 x 1138 nm, with the nanoparticle located at pixel 185 (dashed black line). The differently colored lines in each graph correspond to different time

points during an experiment ranging from incoming high (black lines 4.6 µM) to low fluorescein concentrations (colored line 2.4 µM). The incoming concentration decreased with time during the experiment, which changes the intensity distributions along the nanochannels. A decrease in intensity can be observed downstream of the catalyst particles for all measurements, which can be explained by wall adsorption, desorption and diffusion, as discussed in the main text.

Supplementary Note 6

Varying fluorescein concentration

Supplementary Figure 12a shows the mean fluorescence intensity time evolution for reference channels (average of 15 empty nanochannels) during two similar measurements. As can be seen, as reactants are flushed through the nanochannels from the upstream on-chip reservoir, the intensity decreases slowly during the experiment, probably due to a very slow background reaction on unspecific sites in the microchannels of the nanofluidic device. Since the fluorescence signal obtained in the reference channels is proportional to the fluorescein concentration in solution flushed through the system (Supplementary Figure 12b), it gives an accurate measure for the variation in the incoming fluorescein concentration during the measurement and is thus used to derive the correct concentration value for each time point of an experiment. A control measurement with substantially lower irradiance was also performed to ensure that the observed decrease in fluorescence over time is not due to bleaching or other light induced effects.

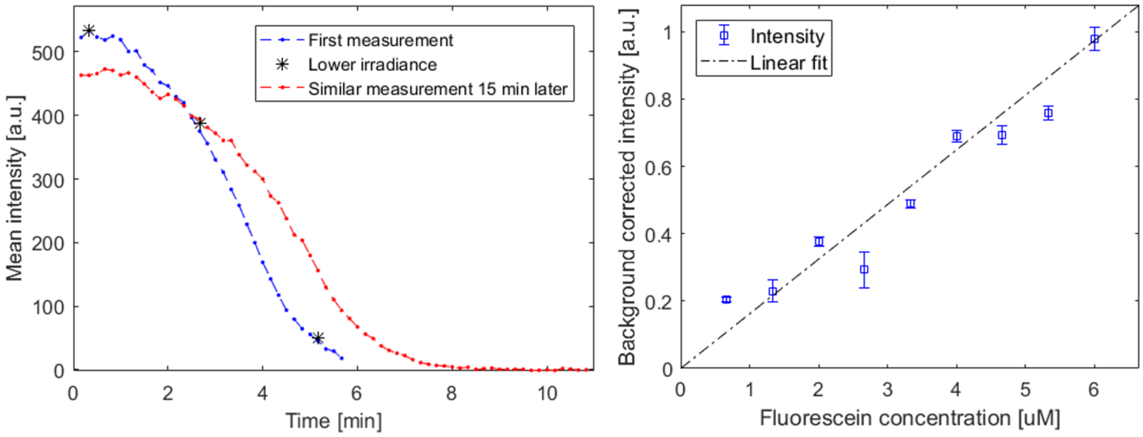

Supplementary Figure 12. a) Measured time evolution of the mean fluorescence intensity (proportional to fluorescein concentration) in 15 empty reference channels, integrated over the region of interest during the duration of a typical experiment. b) Background-corrected intensity of fluorescein concentrations from 0.668 µM to 6.68 µM. The intensity follows a linear trend with respect to fluorescein concentration. The error bars indicate the standard deviation for 10 measured intensity values at each concentration.

Supplementary Note 7

Reaction simulations in 1D

The fluorescein concentration distribution in a nanochannel during reaction was simulated using the convection diffusion equation in 1D:

$$\frac{\partial C}{\partial t} + v\frac{\partial C}{\partial x} = D\frac{\partial^2 C}{\partial x^2}, \tag{2}$$

where C is the concentration v is the flow rate and D is the diffusion constant. Numerically, using the Forward-Time Central-Space method (FTCS)[4] this becomes:

$$\frac{C_j^{n+1} - C_j^n}{\Delta t} + v\frac{C_{j+1}^n - C_{j-1}^n}{2h} = D\frac{C_{j+1}^n - 2C_j^n + C_{j-1}^n}{h^2}, \tag{3}$$

where Δt is a time step and h is a step in space that follows the condition $\Delta t \leq \frac{h^2}{2D}$, n denotes the time point, and j denotes the lateral position. Allowing a reaction at the central position of the nanochannel following the Langmuir Hinshelwood mechanism, using the experimentally derived flow rate (cf. Supplementary Figure 17 & Supplementary Figure 18) and using a value of 425 µm^2 per s for the diffusion constant of fluorescein[5] yields a fluorescein distribution along the nanochannels as depicted in Figure 2b (blue line) in the main text.

By adding a self-equilibrating interaction of adsorption and desorption, as well as molecular diffusion of fluorescein on the nanochannel walls, a similar calculation based on linear adsorption was executed in combination with the FTCS framework. This self-equilibrating interaction was achieved by separating the function for fluorescein concentration in the channels to one concentration on the channel walls (with a lowered diffusion constant) and another function for the concentration in the liquid, both following Supplementary Equation 3 for evolution in time and space. During each iteration of the numerical method, a percentage of the concentration on the channel walls was exchanged with an equal percentage of the concentration in the liquid, such that:

$$C_j^{n+1} = C_j^n \cdot (1 - k_{\text{wall}}) + \text{Cwall}_j^n \cdot k_{\text{wall}} \tag{4}$$

$$\text{Cwall}_j^{n+1} = \text{Cwall}_j^n \cdot (1 - k_{\text{wall}}) + C_j^n \cdot k_{\text{wall}}, \tag{5}$$

where Cwall is the concentration on the walls and k_{wall} is an equilibrium constant. Afterwards, a scaling factor (f_I) was implemented between the concentration on the wall and the concentration in the liquid in order to fit the simulated distribution (as normalized intensity (I_j^n)) to the experimental data:

$$I_j^n = C_j^n + \text{Cwall}_j^n \cdot f_I. \tag{6}$$

To obtain a good fit, the scaling factor should be on the order of 10^1, meaning that this model requires most molecules to be adsorbed to the channel walls. Qualitatively, this description of the system fits well with experimental data and when evaluating the decrease in fluorescence intensity directly after the catalytic particle there is a clear correlation between experiment and simulation (Supplementary Figure 13).

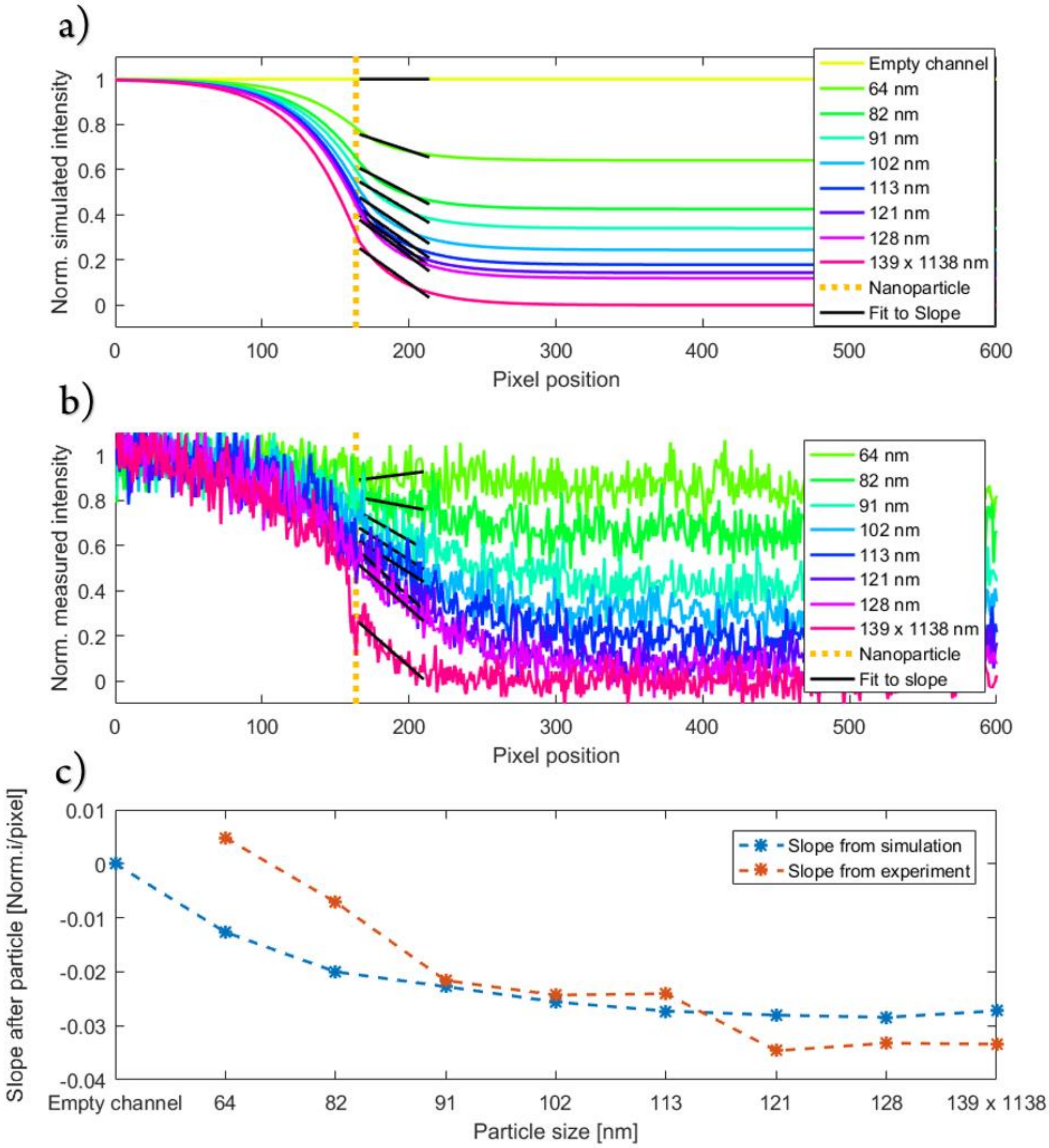

Supplementary Figure 13. Comparison between (a) 1D-simulated intensity distributions with wall adsorption, desorption and diffusion and (b) experimentally measured fluorescence intensity distribution evolutions. The colored lines in (a) and (b) correspond to the different nanoparticle sizes while the black lines are linear fits to the slope in intensity distribution taken directly after the position of the catalytic particle (yellow dotted line). c) Comparison between the slopes in the fluorescence intensity distribution extracted directly after the catalyst particle position for the different particle sizes. Note the good agreement between calculations and experiment.

Supplementary Note 8

Reaction simulations in 3D

The fluorescein concentration distribution in the nanochannel (without wall-adsorption) during reaction was also simulated in 3D, to benchmark the 1D solution presented above. The conversion of a tracer species in a three-dimensional computational domain, representing a 100 nm x 250 nm x 20 μm section of the complete channel, was obtained by solving the coupled system of partial differential equations representing the reactive fluid flow (the Navier-Stokes equations and a convection-diffusion equation for the tracer). The particle was positioned at a streamwise position of 15 μm from the domain inlet. The equation system was discretized in a finite volume framework and solved using the computational fluid dynamics (CFD) code ANSYS Fluent 15.0.7. The no-slip boundary condition was imposed at the wall for the fluid flow, with a specified velocity at the inlet and a specified pressure at the outlet. The tracer concentration field was obtained using a specified inlet mass fraction and zero diffusive flux at the walls, with exception for the particle surface where either a first-order surface reaction (with varying rate constant, *A*) or zero mass fraction (mimicking the mass-transport limit of infinitely fast reaction) was imposed. With this system the catalytic reduction of fluorescein was simulated for several reaction rates, as well as for mass transport limited reaction (Supplementary Figure 14).

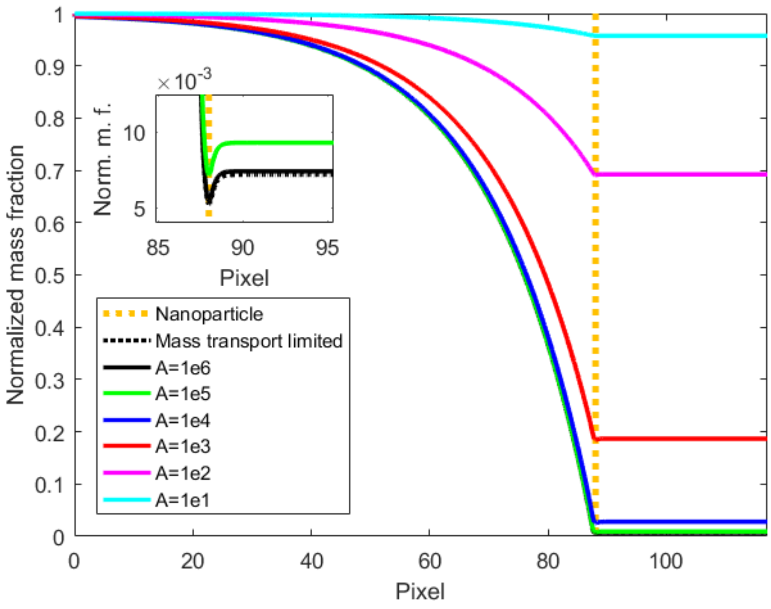

Supplementary Figure 14. 3D-simulated fluorescein concentration distribution along a nanochannel during reaction, calculated for several different reaction rates and for the infinitely fast (mass-transport limited) reaction. The obtained distribution in the channel is very similar to the 1D-simulated displayed in Figure 2c, validating its applicability for our system. The inset shows a zoom in on the particle position to visualize the nearly overlapping mass fractions at high reactivity. The minor increase after the particle is caused by evaluating the mass fraction in the center of the nanochannel (where the reaction rate is high) and mixing with higher concentration from the sides in the channel (where the reaction rate is lower).

Supplementary Note 9
Photobleaching

To minimize photobleaching of fluorescein during our experiments, images were taken every 10 seconds with an exposure time of 100 ms. As a control to assure that bleaching did not affect the calculated ToFs, the following control experiment was performed. We varied the irradiated light intensity between 25 % and 100 % of the maximum available flux and calculated the corresponding normalized ToFs (Supplementary Figure 15). Clearly, within the experimental uncertainty, there is no dependence on irradiance.

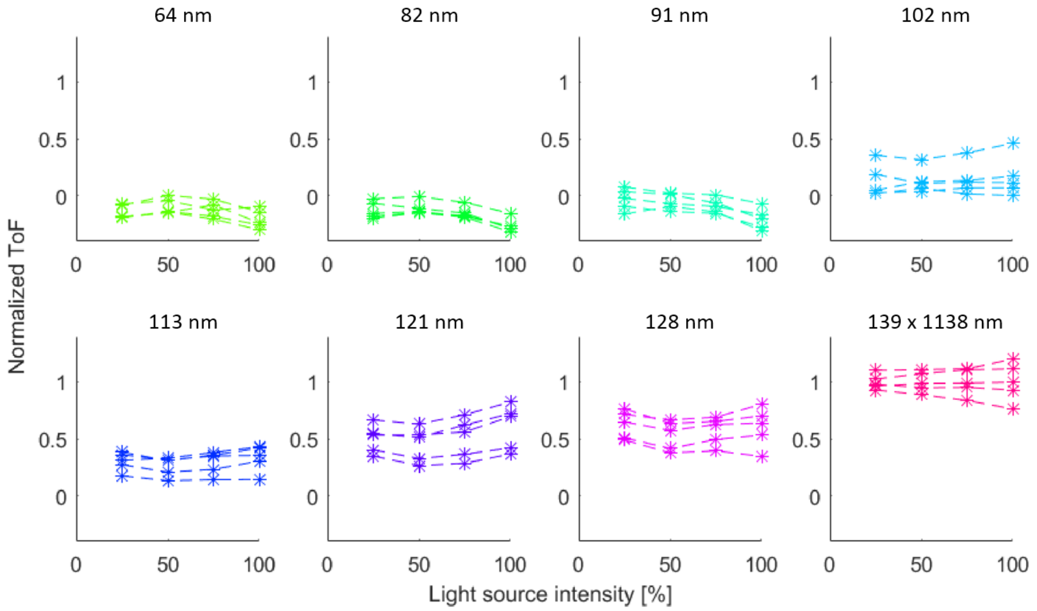

Supplementary Figure 15. Normalized calculated ToF for all particles (grouped according to size in separate panels) on Chip 2 at different light source intensities with respect to the maximal available irradiance. As becomes apparent, the ToF is practically unaffected by light intensity, corroborating that photo bleaching is not affecting the measurement results.

Supplementary Note 10
Pressure drop simulation

To investigate the impact of the presence of a nanoparticle in the nanochannel on the fluid flow, we again employed the 3D simulation scheme (Supplementary Figure 14) to calculate the pressure drop along a 100 nm x 250 nm x 1.3 μm nanochannel decorated with an Au patch of 20 x 139 x 1138 nm dimensions (same dimensions as particle #8 in Figure 1 in the main text) in the middle of the computational domain, as well as the pressure drop along an identical empty nanochannel (Supplementary Figure 16). In both scenarios, the flow was fully developed at the domain inlet. The variation of the gauge pressure as a function of streamwise position in the segment was calculated using area-weighted averages at 100 locations through the domain. The pressure drop over the complete channel (neglecting minor losses at the inlet and outlet) could be obtained from the linear pressure gradient for the empty segment, and a direct comparison with the particle-filled segment yielded the relative increase in the pressure drop from the presence of the particle. This pressure drop past the particle increased with 53 %, which gave a total increase in pressure drop over the whole channel (350 μm) of 0.2 % ($\frac{\text{pressure drop increase} \cdot \text{segment length}}{\text{total nanochannel length}} = \frac{0.53 \cdot 1.3}{350} = 0.2$ %). Hence the effect of the presence of the particle on the flow distribution over the channels is negligible.

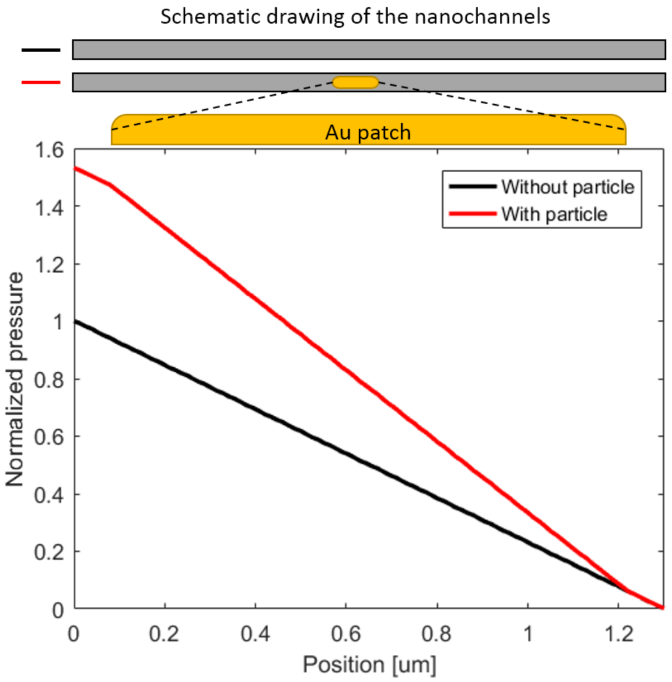

Supplementary Figure 16. Calculated pressure drops over a 100 nm x 250 nm x 1.3 µm empty channel segment (black line) and over the same segment decorated with an 20 x 139 x 1138 nm Au patch in the middle of the segment (red line). The schematic drawing above the graph shows two nanochannels with and without an Au patch and a magnified Au patch to scale with the x-axis. A total increase in pressure drop of 53 % is observed at the position of the particle, which translates into a negligible increase in pressure drop across the whole nanochannel (350 µm), corresponding to only 0.2 %.

Supplementary Note 11

Calculating flow rate

To calculate the ToFs of the individual nanoparticles, the reactant flow rate through the nanochannels needs to be known. To determine the flow, we sequentially flushed fluorescein (100 µM) and water through the nanochannels and measure the velocity at which the fluorescent compound entered or exited the nanochannels (Supplementary Figure 17). This was done by choosing a brightness cutoff (here displayed in percentage of the maximum intensity) and by observing how the fluorescence intensity progressed in the channels from frame to frame. The derivative of the gathered positions then gave the flow speed at any given time (Supplementary Figure 17b). By taking the mean value of the flow speed when the flow was on, we obtained an estimate of the true flow speed. This estimate varies slightly with chosen brightness cutoff due to diffusion (Supplementary Figure 17c). However, by measuring the speed in both possible flow directions and by then taking the mean value, the dependence on the cutoff is minimized (Supplementary Figure 17d). The flow rate was measured at four different applied pressures and a linear dependence between the speed and the pressure was observed. The flow speeds were estimated to be 45, 78, 145 and 214 µm per s for applied pressures of 500, 1000, 2000 and 3000 mbar, respectively. The catalytic measurements presented in the paper were all performed at a pressure of 2000 mbar, corresponding to a flow speed of 145 µm per s.

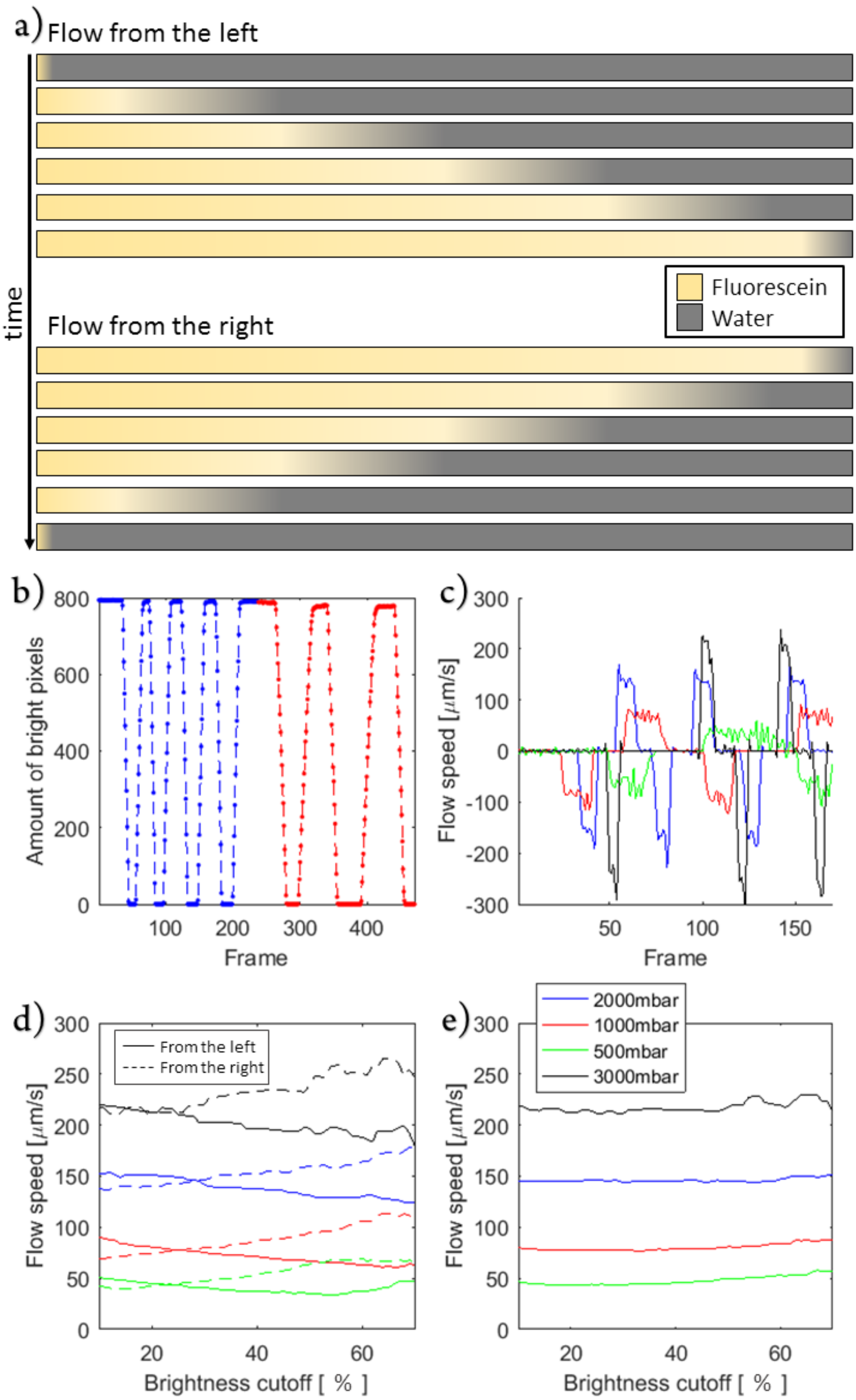

Supplementary Figure 17. Evaluation of sequential flow of fluorescent solution and water in and out of the nanochannels to determine the flow rate at different applied external pressure. a) Schematic of a nanochannel while flowing in fluorescein from the left or water from the right. b) The amount of bright pixels at a brightness cutoff of 50 % for 2000 mbar (blue) and 1000 mbar (red) applied external pressure. c) The derivative of the amount of bright pixels rescaled to correspond to the flow speed in µm per s. d) The flow speeds from the left (solid lines) and flow speeds from the right (dashed lines) at varying brightness cutoffs. e) The mean values of flow speeds from either direction.

Another way to illustrate and estimate the flow speed, and to, in addition, assess the contribution from and amount of diffusion in a nanochannel at the present conditions, is to fit the measured fluorescence intensity along a nanochannel with the 1D convection diffusion equation (Supplementary Equation 2). Using the flow speed estimated above and a value of 425 μm^2 per s for the diffusion constant of fluorescein[5], as well as numerical iterations, the distribution of fluorescein in the channels can be estimated as a function of time, starting from its injection into the channel at t=0 (Supplementary Figure 18). Photo bleaching was also accounted for in the numerical iterations, since the measured steady state distributions decreased in intensity from left to right (yellow lines in Supplementary Figure 18). These simulations fit well with experimental data and prove that the calculated flow rates (Supplementary Figure 17) are correct.

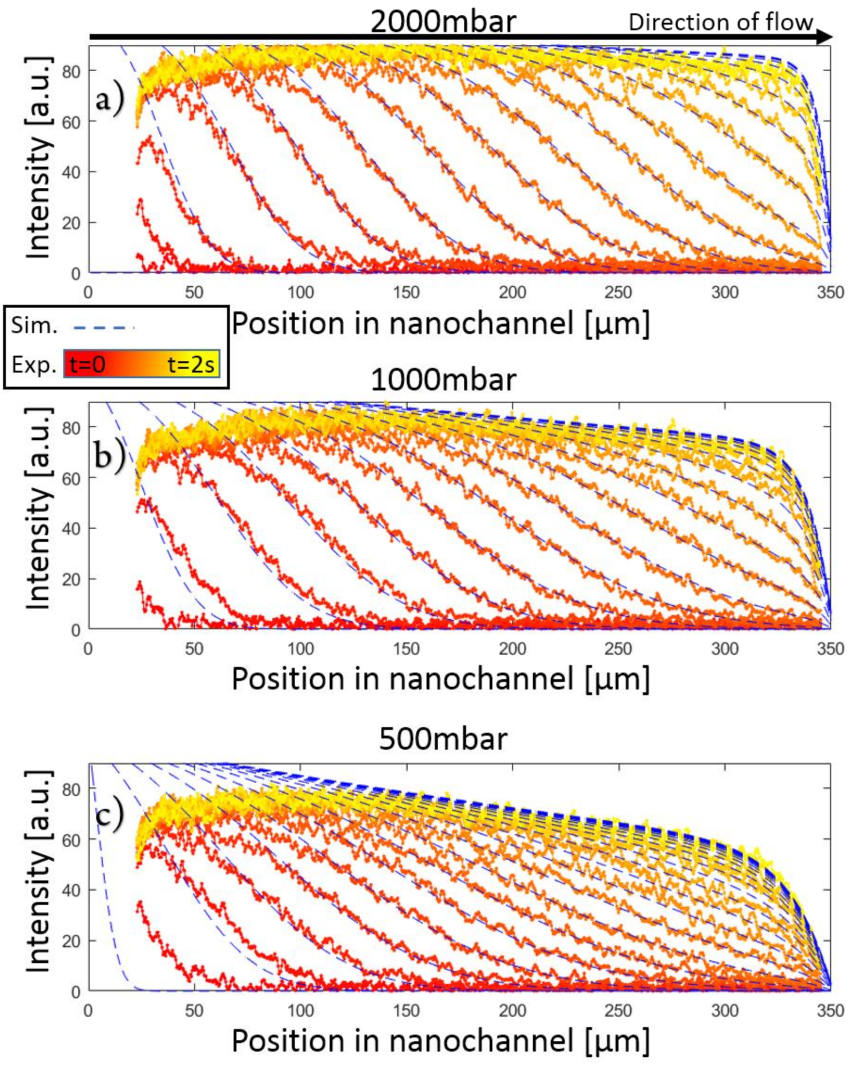

Supplementary Figure 18. Temporal evolution of measured and numerically fitted (based on Supplementary Equation 1) fluorescence intensity distributions along a nanochannel after injection at t=0 for applied external pressures of 2000 mbar (a), 1000 mbar (b) and 500 mbar (c) external pressure. The red-yellow lines are experimentally measured at ten frames per second and the blue dashed lines are the fits based on Supplementary Equation 1.

Supplementary Discussion

Supplementary Note 12

Reproducibility of particle-specific ToF and single particle activity

Supplementary Figure 19, Supplementary Figure 20 and Supplementary Figure 21 display individual and mean ToFs derived for all particles measured on Chip 1 and 2 from three similar experiments, in analogy to the data displayed in Figure 3 in the main text. The same trends and can be observed for all measurements.

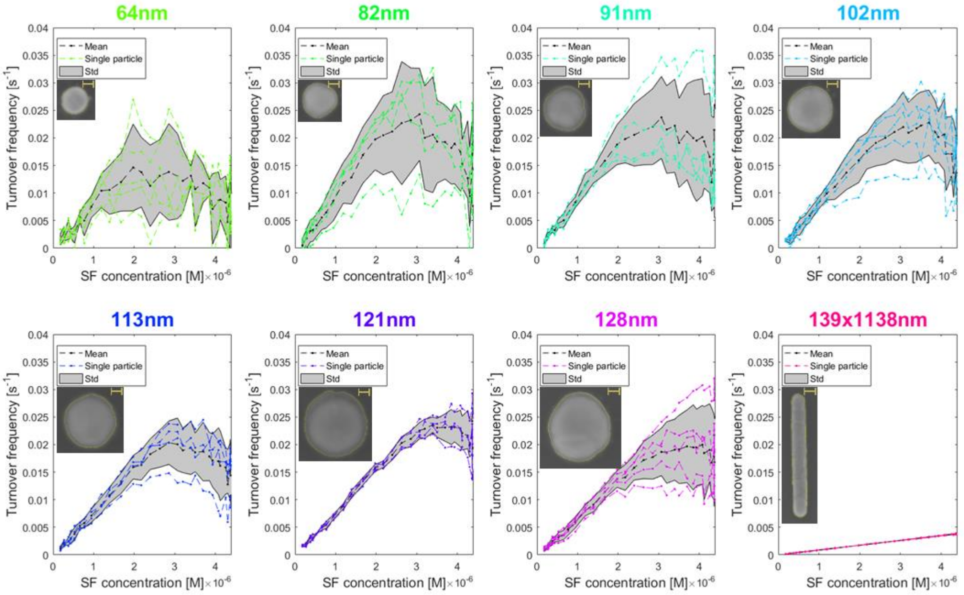

Supplementary Figure 19. Summary of individual and mean ToFs derived for all 32 particles on chip 1 for a specific set of experiments. Titles display particle size and insets show particles imaged with SEM after cleaning. Scale bars are 20 nm in all insets except for the patch with size 139 x 1138 nm, where it is 100 nm. Black and colored lines display mean and individual particle ToFs respectively, and the gray shaded areas depict the standard deviation. The fluorescein concentration was varied between 0 and 4.4 µM and the borohydride concentration was kept constant at 50 mM.

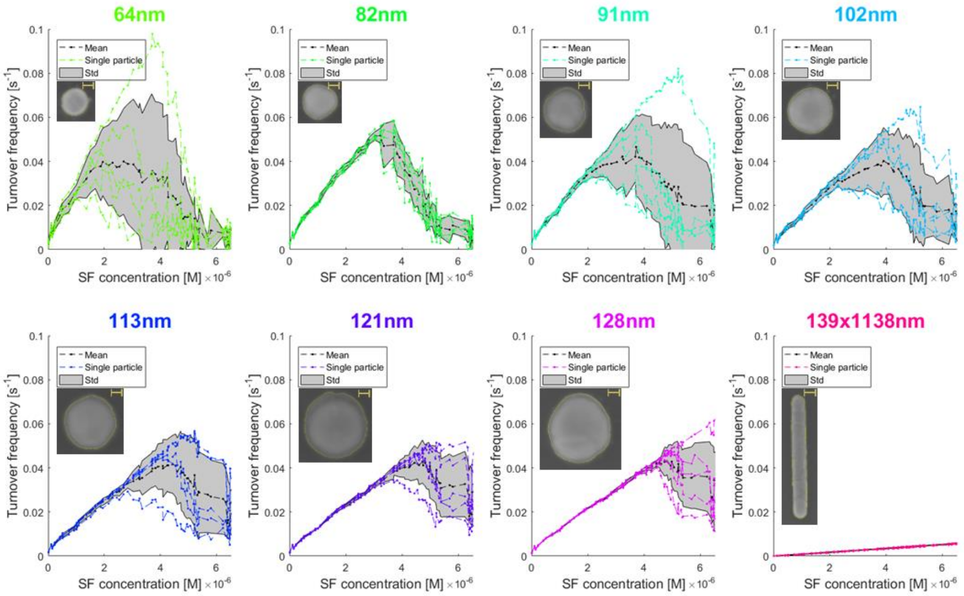

Supplementary Figure 20. Summary of individual and mean ToFs derived for all 35 particles on chip 2 for a specific set of experiments. Titles display particle size and insets show particles imaged with SEM after cleaning. Scale bars are 20 nm in all insets except for the patch with size 139 x 1138 nm, where it is 100 nm. Black and colored lines display mean and individual particle ToFs respectively, and the gray shaded areas depict the standard deviation. The fluorescein concentration was varied between 0 and 6.5 µM and the borohydride concentration was kept constant at 50 mM.

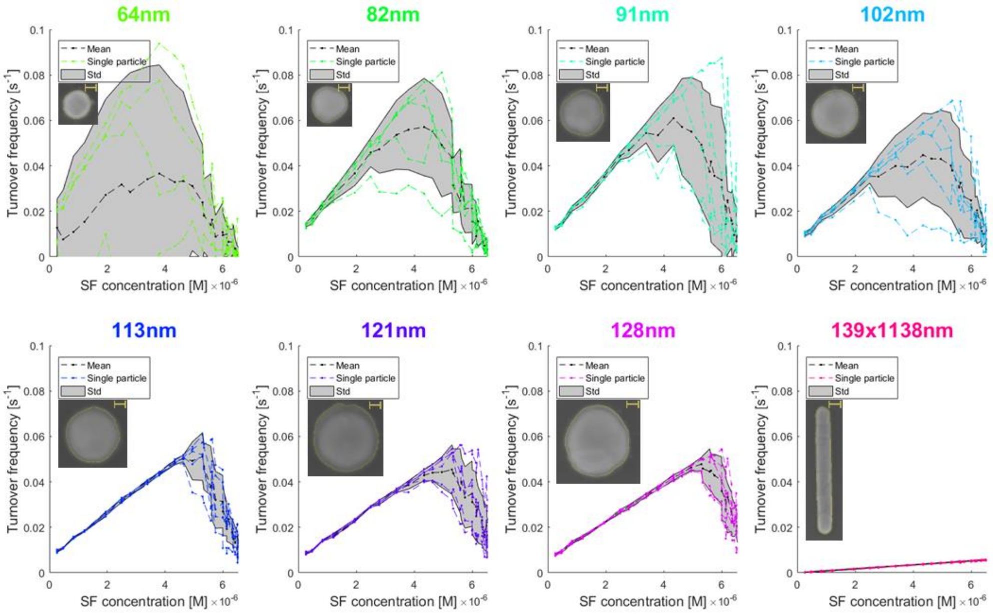

Supplementary Figure 21. Summary of individual and mean ToFs derived for all 35 particles on chip 2 for a specific set of experiments. Titles display particle size and insets show particles imaged with SEM after cleaning. Scale bars are 20 nm in all insets except for the patch with size 139 x 1138 nm, where it is 100 nm. Black and colored lines display mean and individual particle ToFs respectively, and the gray shaded areas depict the standard deviation. The fluorescein concentration was varied between 0 and 6.5 µM and the borohydride concentration was kept constant at 50 mM.

Supplementary Note 13

Reaction in the mass transport limited regime

To further analyze our data for the mass transport limit regime, as well as to investigate the origin of the different apparent fluorescein concentration-dependencies of the ToF slope in this regime (when operating with nominal rather than local fluorescein concentrations) for the different nanoparticle sizes, we compare the experimental slopes with theoretically calculated ones based on Supplementary Equation 1, which in the mass transport regime becomes

$$\mathrm{ToF} = \frac{C \cdot V}{N}, \quad (7)$$

where C is fluorescein concentration, V is the volume flowing past a particle per second, and N is the number of catalytic sites on the particle (Supplementary Figure 22). For all sizes, the experimental slope is in reasonable agreement with the theoretical one for complete mass transport (Supplementary Figure 22b, c). It also becomes apparent that the larger particles are more mass transport limited than the smaller ones and that, therefore, the agreement between experiment and theory improves.

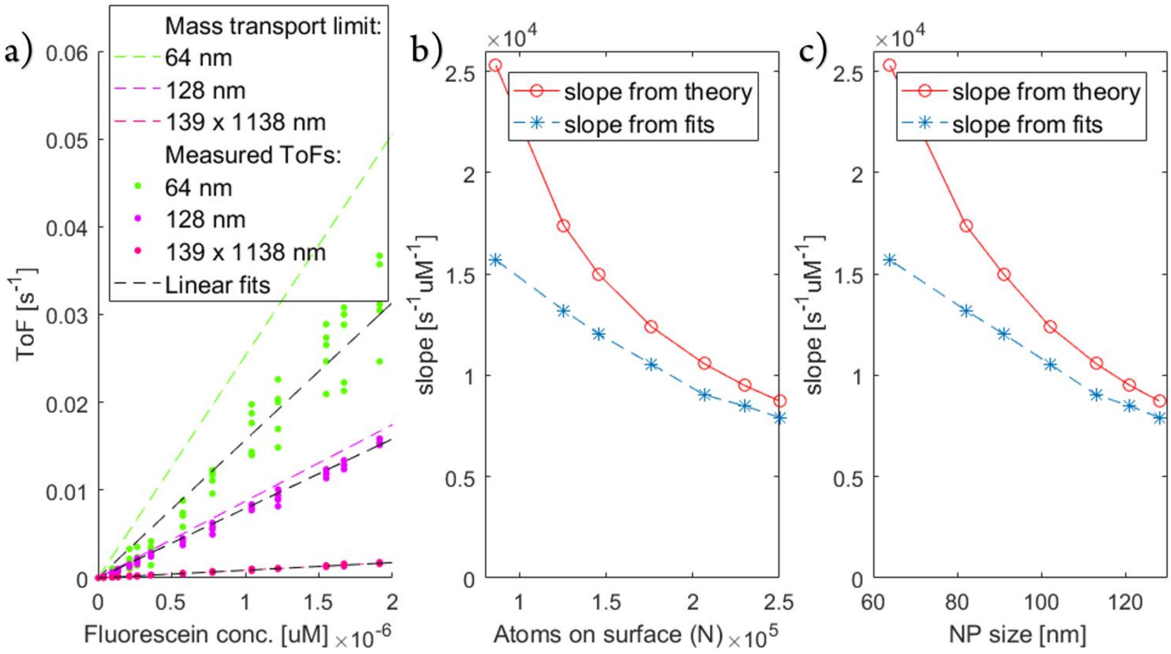

Supplementary Figure 22. Overview of how well the ToF follows the theory for complete mass transport in the mass transport regime. a) Mass transport limit as dashed lines for three particle sizes (64 nm (green), 128 nm (purple), 139 x 1138 nm (pink)) and measured ToFs (dots) as a function of nominal fluorescein concentration. b) Theoretical (red) and experimental (blue) slope for a mass transport limited nanoparticle for the surface areas corresponding to the particle sizes used (Figure 1b). c) The same values as in (b) but as a function of nanoparticle size. In both (b) and (c) the slope for experimental values are close to but lower than theoretical values, meaning that the particle activity is governed mainly but not completely by mass transport, even in the mass transport limited regime.

Supplementary Note 14

Understanding ToF in terms of mass transport limit and surface reaction limit

As discussed in the main text, when displaying the ToF as a function of the experimentally determined local fluorescein concentration at the position of the nanoparticle (Figure 4 and Supplementary Figure 23), the ToFs align at low concentrations for all particle sizes. However, at higher fluorescein concentrations (above ~2 µM) the reaction on most particles is no longer mass transport limited and, thus, the ToFs start to diverge and spread, depending on particle specific reactivity. Assuming a

Langmuir-Hinshelwood reaction mechanism, in which two reactant molecules need to first adsorb and find each other on the surface in order to react and then desorb, in this regime the ToF can be approximately described by the equation[6]

$$\mathrm{ToF} = k \cdot C_S^2 \frac{K_1 K_2 C_1 C_2}{(1+K_1 C_2 + K_2 C_2)^2} \quad (8)$$

where k is the reaction rate, C_S is the total number of sites, $C_{1,2}$ are the reactant concentrations and $K_{1,2}$ are the reactant adsorption constants. An example with k and $K_{1,2}$ chosen to approximately fit the experimental data shows how the reaction becomes surface reaction limited at high fluorescein concentrations (Supplementary Figure 23). Since the activity of each particle is different in the surface reaction limited regime, each particle should have its own upper surface reaction rate limit, based on its own k-value.

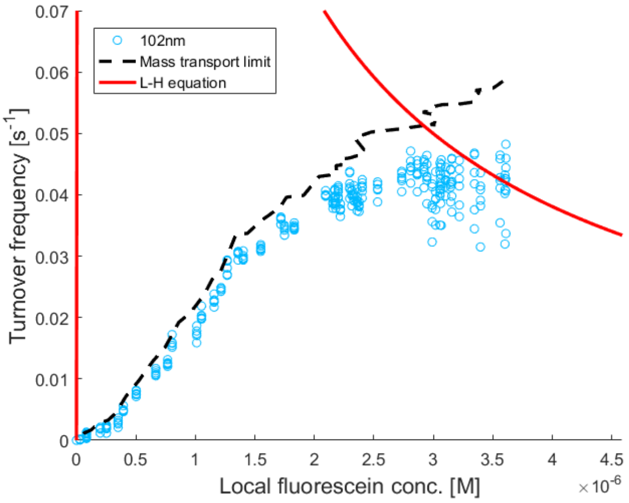

Supplementary Figure 23. The ToFs for five 102 nm particles plotted as function of local fluorescein concentration. The calculated mass transport limited ToF is depicted by the dashed black line and the surface reaction limit according to the Langmuir-Hinshelwood mechanism is depicted by the red line.

Supplementary Note 15

Compilation of all ToFs

Measurements were performed on two nominally identical chips (chip 1 and chip 2) at multiple occasions to investigate to what extent the experiments in general, and the obtained single-particle-specific activities in particular, were reproducible. To this end, we found that the calculated ToF obtained on different days and for different reactant concentrations could vary substantially from experiment to experiment but still yield ToFs of the same order of magnitude. Furthermore, and most importantly, we also found that the activity trends within a sample and between particles were consistent across all experiments (Supplementary Figure 24). Hence, the found variations in absolute activity are most likely due to degradation of borohydride in water, rendering slightly different reaction conditions from experiment to experiment.

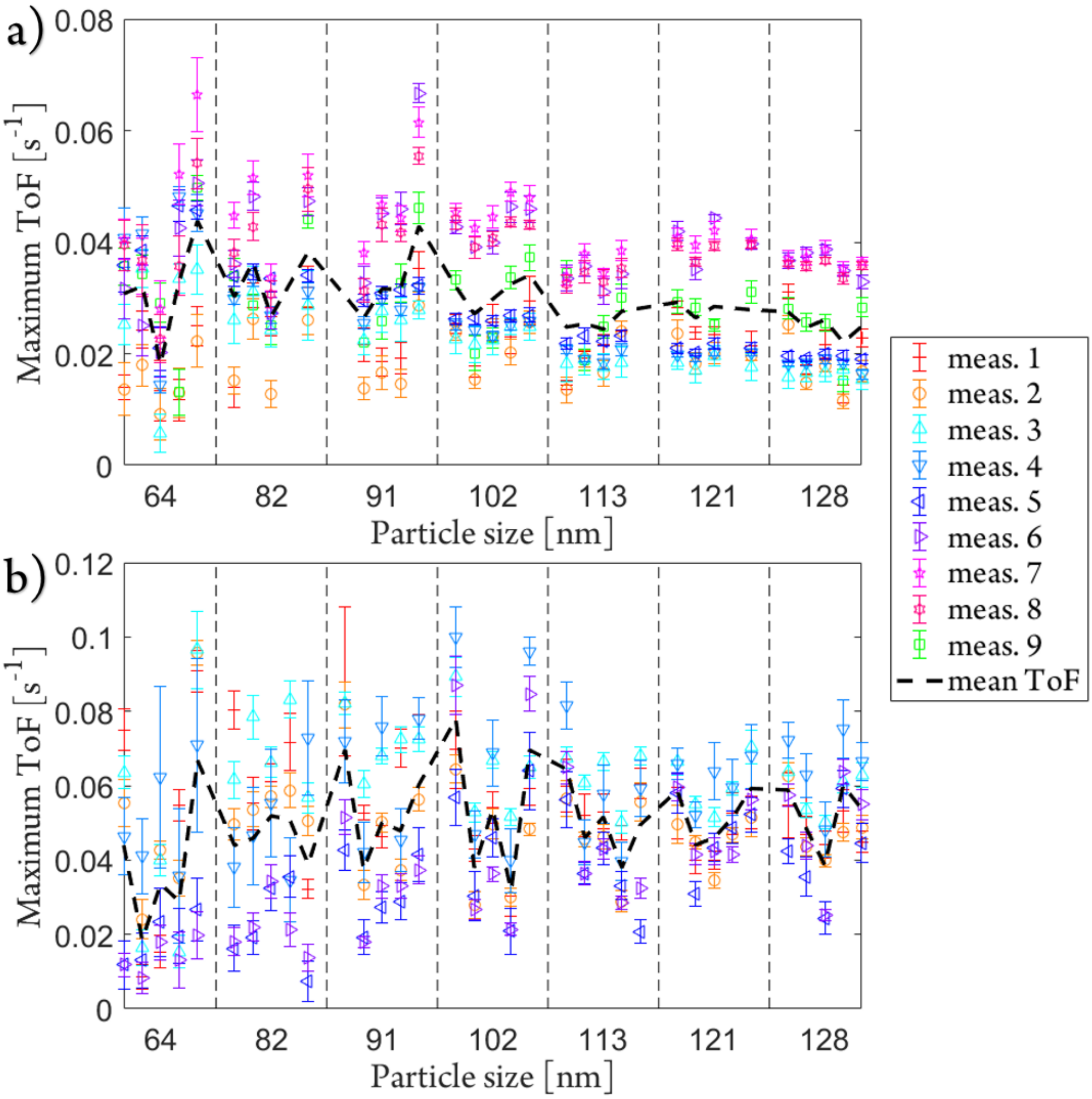

Supplementary Figure 24. (a) Derived maximum ToFs for all particles and all measurements on Chip 1. (b) Derived maximum ToFs for all particles and all measurements on Chip 2. Each color corresponds to a different measurement and the dashed black lines show the mean ToF value for all measurements.

Supplementary Note 16

Comparing surface area variations with variation in particle activity

To estimate the relative contribution of surface area variations between individual nanoparticles (Supplementary Figure 2) and the derived maximal ToFs (Figure 5a), we scaled the surface area calculated for each particle based on TEM image analysis to the same mean value as the one for all maximum ToFs from three measurements in chip 1. When comparing the data (Supplementary Figure 25a and b), it becomes apparent that the variations in maximum ToF are at least one order of magnitude larger than the variations in surface area. From this we conclude that the variations in ToF cannot be attributed only to variations in particle surface area.

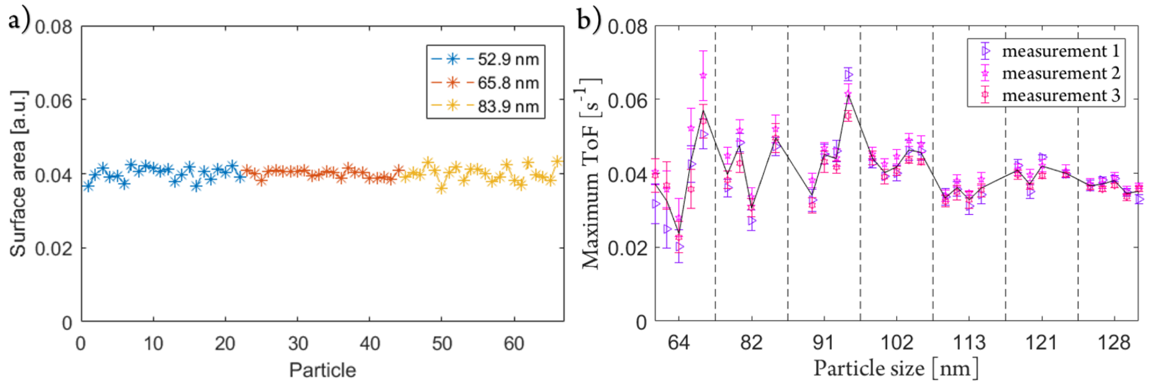

Supplementary Figure 25. (a) Scaled surface areas calculated based on TEM images of 66 nanoparticles (three different nominal sizes as depicted in the legend). (b) Maximum ToFs derived for 32 nanoparticles. The surface area in (a) is scaled to have the same mean value of 0.04 as the mean maximum ToF in (b) to be able to visualize a possible correlation.

Supplementary Note 17

Correlation between ToF measurements

The correlation coefficients for different maximum ToF measurements were calculated to demonstrate the reoccurring trends in a more mathematically robust way. To do this, we first subtracted the mean ToF value for each particle size from the individual ToFs for each set of particle sizes (Supplementary Figure 26). It is clear from these graphs that some trends are retained through all measurements. To obtain a value for how strongly the measurements correlated, correlation coefficients were calculated according to:

$$\text{correlation coefficient} = \frac{\text{Cov}(f,g)}{\sigma_f \cdot \sigma_g} \tag{9}$$

Where f and g are arrays of measurement values with the mean value for each size subtracted (i.e. the values in Supplementary Figure 26). A clear correlation is visible between all measurements done in chip 1 (Supplementary Figure 27). Correlation coefficients of 0.62, 0.58 and 0.22 were calculated as the mean for all correlations between measurements in chip 1, chip 2 and chip 1 correlated with chip 2, respectively (Supplementary Figure 28).

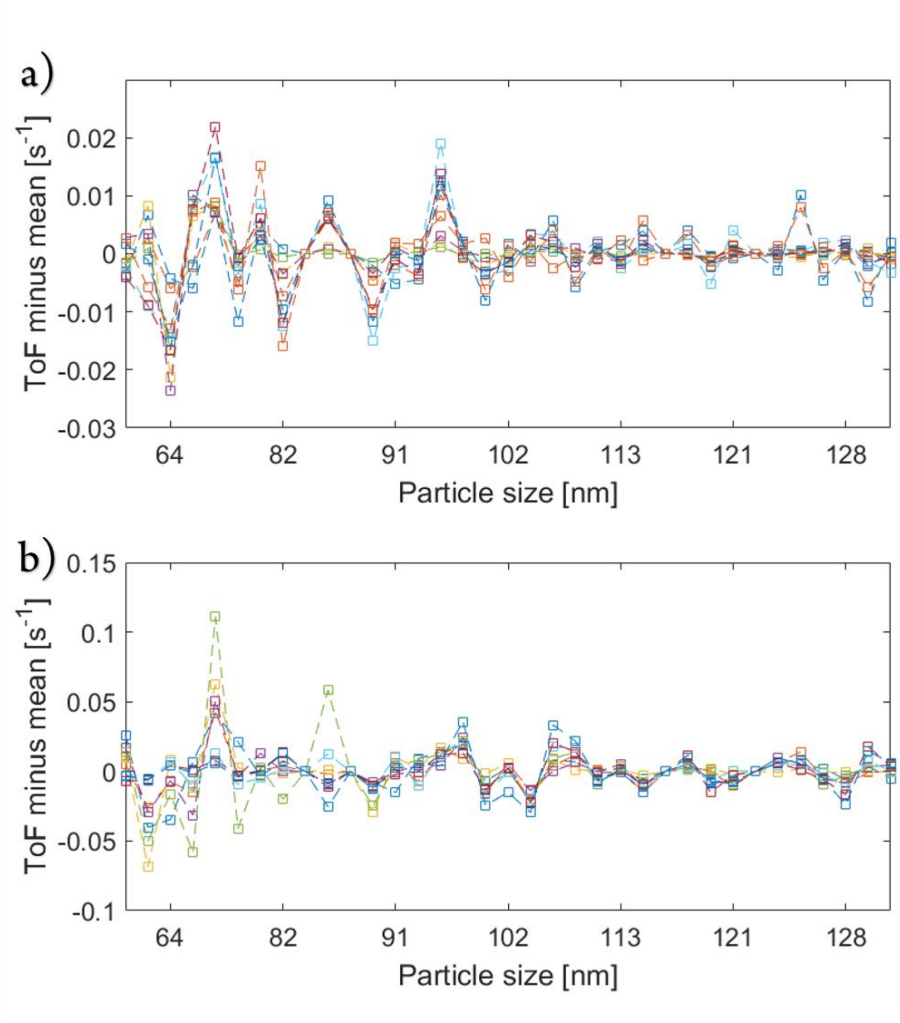

Supplementary Figure 26. ToFs minus mean ToF value for each particle size and for all executed measurements (color code) in a) chip 1 and b) chip 2.

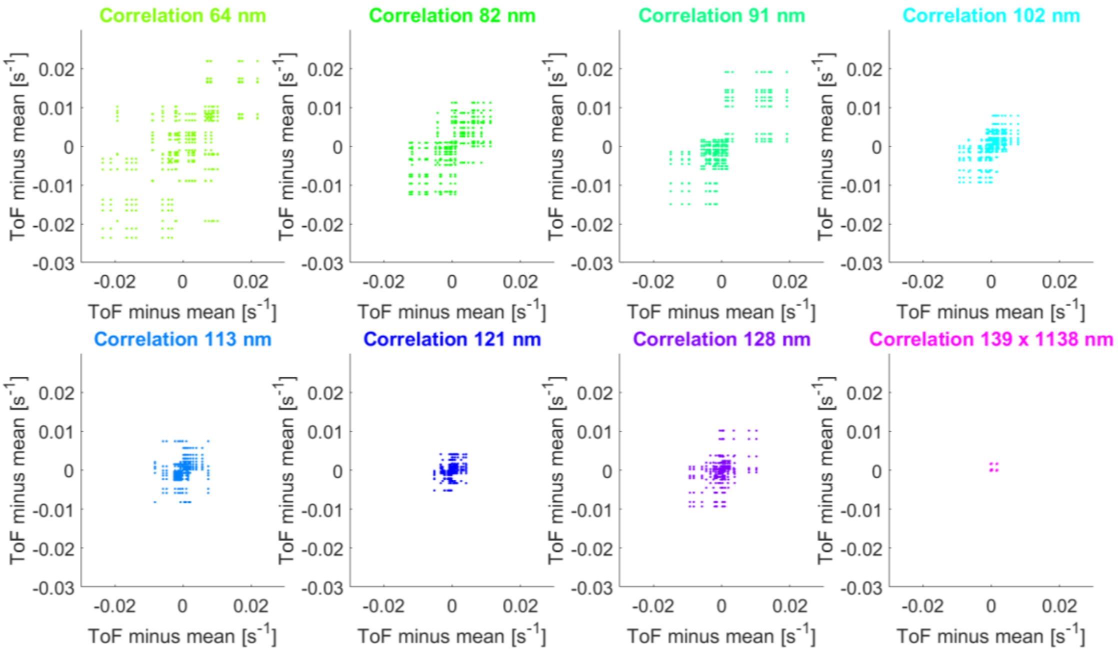

Supplementary Figure 27. Scatter plots to visualize correlations between individual particle activities from measurements in chip 1. Note that the individuality in reactivity is stronger for smaller particles.

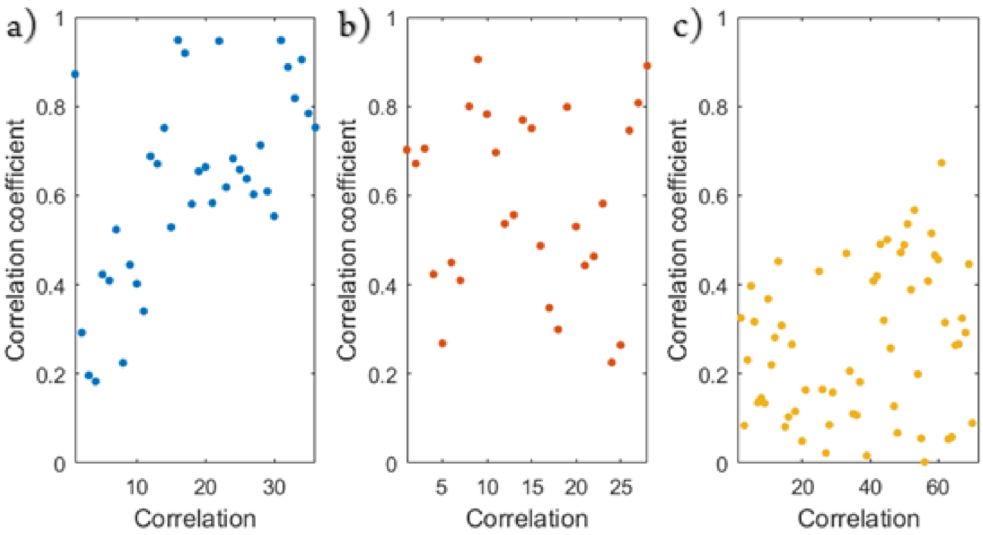

Supplementary Figure 28. Correlation coefficients for all measurements in a) chip 1, b) chip 2 and, c) chip 1 correlated with chip 2.

Supplementary Note 18

Amplex ultra red oxidation

With our platform, we are also able to measure the catalytic activity from a fluorescence "turn-on" reaction where a non-fluorescent reactant forms a fluorescent product. To illustrate this here in a preliminary fashion, we used the oxidation of amplex ultra red to resorufin by hydrogen peroxide. Our reactions were performed at concentrations of 0.25, 0.5, 1 and 1.8 mM amplex ultra red and 90mM hydrogen peroxide in water of pH 11, we were able to measure ToFs of up to 0.01 s^{-1} for single nanoparticles (Supplementary Figure 29). We then evaluated the data in a similar fashion as for the

reduction of fluorescein (compare Supplementary Figure 29 with Supplementary Figure 10). As the main difference, we determined the rates by using a series of reference measurements to correlate fluorescence intensity with resorufin concentration.

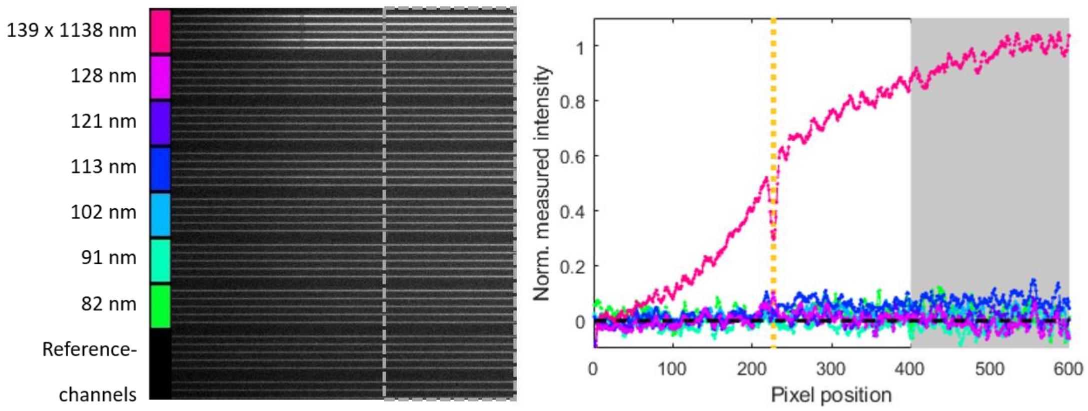

Supplementary Figure 29. The left image shows a representative CCD image of a turn on experiment with color-coded nanochannel sets that contain Au particles of a certain size. The image to the right shows the normalized mean fluorescence intensity profile along each set of channels containing a certain particle size. The dashed gray area in the left image corresponds to the shaded gray area in the graph to the right and is the region of interest located downstream of the nanoparticles. The yellow dashed line displays the nanoparticle positions.

As a complicating factor for this system, amplex ultra red is known to photo-autocatalytically oxidize and in this way produce additional resorufin. To minimize the effect of this unwanted side reaction, very low light exposure is needed.[7,8] Therefore, all measurements were performed at low light intensity with 200 milliseconds exposure time and 5 seconds between each image. The correspondingly obtained ToFs per particle as a function of amplex ultra red concentration are displayed in Supplementary Figure 30a-b. To ascertain minimal influence of the background reaction, we executed additional experiments at half flow rate (obtained by applying 1000 mbar flow pressure to the fluidic system as opposed to 2000 mbar) resulting in doubling of the produced resorufin concentration downstream of the nanoparticles. Specifically, when effectively doubling the concentration downstream of the particles by applying half the flow rate, the measured fluorescence signal increases by a factor two (Supplementary Figure 30c-d) displaying a linear scaling in the evaluated signal. In combination with the linear scaling of the fluorescence signal as a function of light intensity, this indicates that the autocatalytic photo-oxidation of amplex ultra red is efficiently suppressed.

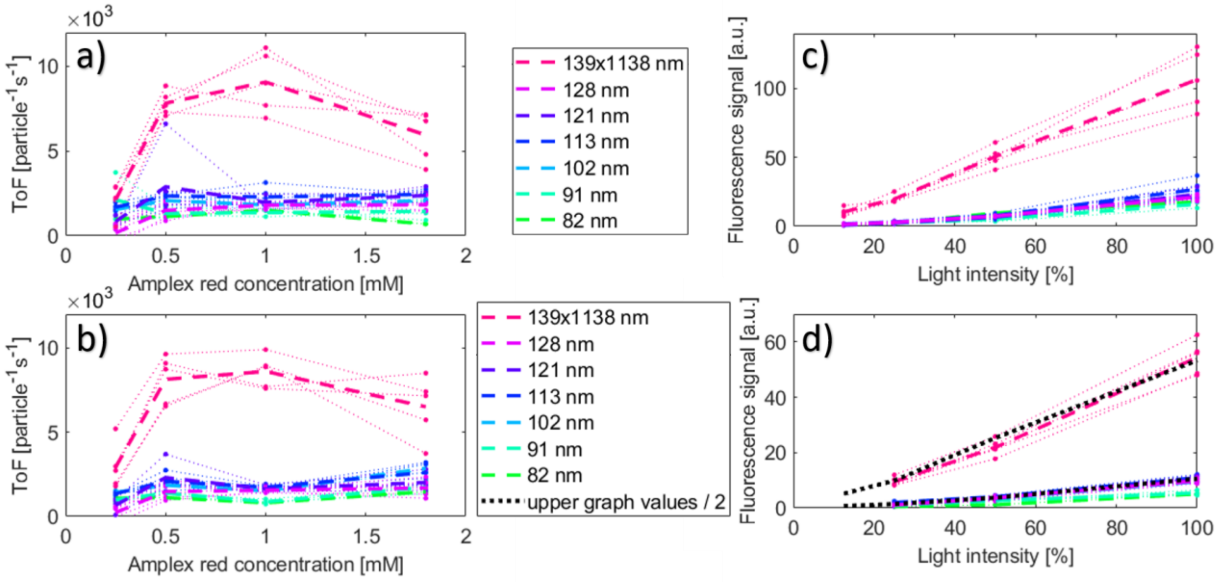

Supplementary Figure 30. Measurements of catalytic amplex ultra red oxidation to resorufin over Au nanoparticles of different size. Colored dotted lines correspond to individual particles, dashed lines are the mean values for each size and black dotted lines show the mean values for the two largest particles sizes in the top graphs divided by 2. Measurements were done at (a) 1000 mbar flow pressure, 90 mM hydrogen peroxide and 100% light intensity, (b) 2000 mbar flow pressure, 90mM hydrogen peroxide and 100% light intensity, (c) 1000 mbar flow pressure, 90 mM hydrogen peroxide and 1 mM amplex ultra red, (d) 2000 mbar flow pressure, 90 mM hydrogen peroxide and 1 mM amplex ultra red. Note that the values at double flow pressure, i.e. doubled reactant flow rate through the nanochannels, results in half the fluorescence intensity.

Supplementary References

1 Grant, A. W., Hu, Q. H. & Kasemo, B. Transmission electron microscopy windows for nanofabricated structures. *Nanotechnology* **15**, 1175-1181, (2004).

2 Fritzsche, J. *et al.* Single Particle Nanoplasmonic Sensing in Individual Nanofluidic Channels. *Nano Lett.* **16**, 7857-7864, (2016).

3 Zorić, I., Zäch, M., Kasemo, B. & Langhammer, C. Gold, Platinum, and Aluminum Nanodisk Plasmons: Material Independence, Subradiance, and Damping Mechanisms. *ACS Nano* **5**, 2535-2546, (2011).

4 Pletcher, R. H., Tannehill, J. C. & Anderson, D. A. *Computational fluid mechanics and heat transfer*. Third edition. edn, (CRC Press, Taylor & Francis Group, Boca Raton, 2013).

5 Culbertson, C. T., Jacobson, S. C. & Ramsey, J. M. Diffusion coefficient measurements in microfluidic devices. *Talanta* **56**, 365-373, (2002).

6 Somorjai, G. A. & Li, Y. *Introduction to surface chemistry and catalysis*. 2nd ed. edn, (John Wiley & Sons, Hoboken, 2010).

7 Mayer, K. M., Shnipes, J., Davis, D. & Walt, D. R. Catalytic kinetics of single gold nanoparticles observed via optical microwell arrays. *Nanotechnology* **26**, 055704, (2015).

8 Zhao, B., Summers, F. A. & Mason, R. P. Photooxidation of Amplex Red to resorufin: implications of exposing the Amplex Red assay to light. *Free Radic. Biol. Med.* **53**, 1080-1087, (2012).
