# Supplementary material for: A nanofluidic device for parallel single nanoparticle catalysis in solution
Source: Nat Commun. 2019 Sep 27;10:4426. doi: 10.1038/s41467-019-12458-1 (PMC6764984; doi:10.1038/s41467-019-12458-1)
Supplement: Supplementary file 2 — Peer Review [file 41467_2019_12458_MOESM2_ESM.pdf]

Reviewers' comments:

Reviewer #1 (Remarks to the Author):

This manuscript presents a nanofluidic device for highly parallelized single nanoparticle catalysis in solution based on fluorescence microscopy. The authors fabricate the Au nanoparticles ranged in size from 64 nm to 128 nm in diameter inside their corresponding individual nanochannels through electron-beam lithography. The fluorescence intensity along a channel for each particle is detected by a CCD, where the brightness of each channel corresponds to the fluorescein flowing through the fluidic system and the decreased emission intensity is due to the reduction of fluorescein by Au nanoparticles in the channel with NaBH4. Further, the work provides detailed quantitative analysis of the reaction in each channel, such as the turnover frequencies (ToFs) of individual nanoparticle, particle-specific ToF as a function of location reactant concentration, and the trends in particle-specific activity. As such, the matter is of interest; however there are some issues need to be addressed before its publication:
1. Line 130-140, P5 in main text: Discussion the decreased fluorescence intensity downstream the catalyst, the authors attribute it to the fluorescein binding to the inner walls. However, fluorescence intensity along empty reference channels does not decrease seen from the Figure 2b. This point needs more interpretation.
2. Only 5 Au nanoparticles in each size were analyzed during experiment. I would suspect the accuracy of catalytic activity (or ToFs) for per particle size estimated in this study. In other words, Experimental data may not be general. Generally, the number of each type of particle should be large enough (at least > 10) to reach a reliable statistic results.
3. P14 in supporting information: this section demonstrates that the fluorescence signal is proportional to the fluorescein concentration in the reference channels, where the authors should add experiments or literatures to confirm it. Additionally, in Au decorated channels, the concentration value of each time point is derived from the fluorescence intensity, however, the fluorescence intensity along the channel is affected by many factors. Thus, the quantitative relationship between the fluorescence intensity and fluorescein concentration should be provided.
4. Line 174-178, P7 in main text: the authors should add more explanations and literatures to prove that the reaction is mostly mass transport limited and never reach the surface reaction limited regime. In addition, the sentence “This can be understood as that we for the largest …” should be changed to “This can be understood as that for the largest …”
5. Line225-228, P9 in main text: more literatures should be supplied to confirm the conclusion that the spread activity of individual gold nanoparticle for a certain size can be assigned to the different abundance of low coordination and defect sites imposed by the microstructure.
6. Line 269, P11 in main text: “increasing linearly from ca. 0.1 – 0.3 s-1 per site” should be “increasing linearly from ca. 0.01 – 0.03 s-1 per site” according to the Figure 4.
7. There are two SEM images missing in Figure SI 4d.

Reviewer #2 (Remarks to the Author):

In their manuscript Levin et al. have demonstrated the use of nanofluidic device for parallel analysis of the catalytic reactivity of single nanoparticles in solution. The main advantages of the experimental setup presented by the authors are: 1. Simultaneous analysis of particles reactivity under identical reaction conditions and 2. Conducting reactivity measurements under realistic reaction conditions. The authors have studied with their setup the influence of particles’ size on the reactivity under various reactants concentrations. They have identified that as the particles’ size is increased beyond 100 nm, the reactivity is less influenced by changes in reactants concentrations and is less sensitive to variations in the surface properties of the nanoparticles.
The authors describe an interesting study about size-dependent reactivity, which they identified based on single nanoparticles measurements in a nanofluidic setup. However, this experimental approach leads to major limitations in the scientific knowledge that can be extracted, as detailed below:

1. The authors present a different approach for fluorescence microscopy measurements of catalytic reactions, which is in a way opposed to the mainstream approach. The common way to use fluorescence microscopy measurements is by probing the formation of fluorescent products and then by using super-resolution techniques, identifying the site-dependent reactivity with a resolution of < 10 nm (see for example, JACS 132 (1), 138-146 and ACIE 48 (49), 9285-9289). The high spatial resolution is achieved by using very low reactants concentration – thus enabling the detection of individual reactions and mapping site-dependent reactivity on single nanoparticles.
The authors' approach for detection of catalytic reactions limit their spatial resolution to the diffraction limit of light (~ 200 nm), which is larger than most of the particles studied in this work. At this spatial resolution, the authors could have used micro-Raman measurements to map the catalytic transformation in their nanofluidic reactor with some payment in the spatial resolution (500 nm) but with a much richer chemical understanding of the catalytic reaction. Raman micro-spectroscopy measurements with CCD detectors can provide simultaneous detection of the reactivity on multiple nanoparticles.
By tracking the transformation from a fluorescence reactant to a non-fluorescence product the authors have lost the benefits of super-resolution microscopy while using a probing technique that provides very limited molecular understanding of the reaction. Therefore, it is not clear to me that the model reaction and the probing technique (fluorescence microscopy) can effectively address the scientific question that the authors have raised.
2. In their manuscript, the authors describe the current limitations in state-of-the-art florescence microscopy as: 1. low throughput and 2. low reactant concentrations and/or reaction rates. This statement is not accurate. Fluorescence microscopy measurements were previously performed on large scale, covering the reactivity of hundreds of nanoparticles (ACS Catal. 2013, 3, 1448-1453). The use of low reactants concentration was specifically chosen in fluorescence measurements in order to reach super-resolution conditions.
3. The authors have detected that as the size of the particles is increased there is less variation in the particles reactivity and the reactivity is not limited by surface-reaction. The authors have correlated this observation with differences in the density of low-coordinated surface sites. As I see it, this correlation needs to be further validated:
a. The authors show that the reactivity is widely changed once the particle size is increased from 64 to 100 nm. These particles are already very large (in comparison to highly reactive Au nanoparticles, which are in the size range of <10 nm). Why are there such vast differences in the reactivity of particles in this specific size range? Are there any clear evidence for differences in the density of low coordinate surface atoms within the probed particles? I would assume that annealing (500-600 C) of the smaller particles would result a decrease in their density of low-coordinate surface sites – was this experiment done? What is the influence of such pretreatment on the reactivity of the various nanoparticles?
b. The authors have shown that during the preparation of Au particles there were few smaller particles that were formed and were washed away by various pretreatments. Did the authors conduct HR-SEM measurements to identify that there are no small (> 10 nm), highly reactive Au nanoparticles on the surface? Did the authors check for any leaching and presence of Au residues in the products solution?
c. The authors use the fluorescence quenching as a marker for reactant-to-product transformation. Additional experiments are needed in order to molecularly identify the product and verify that the non-fluorescence product is the major/only product that is formed in the reaction.
d. The authors have shown that for small particles as the fluorescence reactant concentration is increased the reactivity is decreased. This indicates that the reaction is controlled by Langmuir-Hinshelwood mechanism and not by Eley–Rideal mechanism. The authors should verify that experimentally, by for example changing simultaneously the concentration of the two reactants.

Reviewer #3 (Remarks to the Author):

This research article, by Langhammer, Westerlund and co-workers, reports on the development of a

nanofluidic device, which in combination with fluorescence microscopy allows to investigate metal nanoparticles in various shapes ands sizes to assess the interparticle heterogeneities in terms of catalyst activity. For this purpose, the authors have selected the already explored catalytic reduction of fluorescein by borohydride on Au nanoparticles. One of the conclusions is that the setup allows to investigate the TOF of the Au nanoparticles and this number is in the range of 0.1 s-1 per site.

The strength of the paper is its catalyst chemical engineering aspects, in which the authors explore the regimes between diffusion/mass transport control up to surface reactivity control; hence they use their new device to explore what it can learn in real-life catalytic applications. However, on the other hand, one has to recognize that the reactions proposed are father exotic and far away of a real life catalytic system (in terms of metal nanoparticle size; as well as reaction conditions and reaction system). That is the weak part; as one of course wish to see how it can be applied in a broader sense. What I also miss is the comparison with e.g. Raman/SERS type approaches for monitoring in nanofluidic devices the reaction processes. This offers insight in the reaction course as the reaction products and reactants and intermediates can be assessed via the spectra; what is not possible with the fluorescence microscopy approach (or at least not applied in this system).

I am charmed by the mathematical approaches taken in this work; that is the strength of the work. An example being Figure 2c; here one can also see what is possible and that is evident from figure 3. Figure 5 is in my opinion not very clear and the text surrounding this figure can be better written. The concept of single particle catalysis should also be better explained and stressed. As a side remark, as pointed on page 2, the work described is not operand but in situ work. I also not that the references are not well written; e.g. ref. 5, article number is missing; ref. 32, reference not correctly written; and ref. 28, reference not correctly written; but there are more errors like this.

What I miss in the paper is the general applicability (as mentioned already above) and would here like to see an expansion to another reaction and/or another catalyst system; to show what can be done with this nano-fluidic device next to what the authors already discuss. Currently, it remains a showcase for what can be done, but not really much new catalytic chemistry has been learned. Again, it is a very nice chemical engineering approach to the concepts of single particle catalysis and the authors have devised and constructed a nice setup, however, illustrating it potential with a rather well known, and in some way a boring chemical reduction reaction.

Having said all this, the work is to the best of my knowledge novel, and also deserves to be published in a journal such as this one; hence I recommend publication after revisions as outlined above.

Reviewer #1 (Remarks to the Author):

This manuscript presents a nanofluidic device for highly parallelized single nanoparticle catalysis in solution based on fluorescence microscopy. The authors fabricate the Au nanoparticles ranged in size from 64 nm to 128 nm in diameter inside their corresponding individual nanochannels through electron-beam lithography. The fluorescence intensity along a channel for each particle is detected by a CCD, where the brightness of each channel corresponds to the fluorescein flowing through the fluidic system and the decreased emission intensity is due to the reduction of fluorescein by Au nanoparticles in the channel with NaBH4. Further, the work provides detailed quantitative analysis of the reaction in each channel, such as the turnover frequencies (ToFs) of individual nanoparticle, particle-specific ToF as a function of location reactant concentration, and the trends in particle-specific activity. As such, the matter is of interest; however there are some issues need to be addressed before its publication:

Our reply: We thank the Reviewer for the generally positive assessment of our work.

1. Line 130-140, P5 in main text: Discussion the decreased fluorescence intensity downstream the catalyst, the authors attribute it to the fluorescein binding to the inner walls. However, fluorescence intensity along empty reference channels does not decrease seen from the Figure 2b. This point needs more interpretation.

Our reply: The reason why the fluorescence intensity in the reference channels does not change is simply because, there, no fluorescence is quenched by the reaction and the system is in *the same* equilibrium with respect to molecules on the wall and in solution along the whole channel.

2. Only 5 Au nanoparticles in each size were analyzed during experiment. I would suspect the accuracy of catalytic activity (or ToFs) for per particle size estimated in this study. In other words, Experimental data may not be general. Generally, the number of each type of particle should be large enough (at least > 10) to reach a reliable statistic results.

Our reply: We are not sure if we correctly understand what the Reviewer has in mind here because the second sentence is a bit unclear. We believe that the Reviewer is saying that 5 particles per size does not allow unambiguous conclusions about size dependent activity. This is a correct comment, but at the same time it is not the aim of our study to absolutely identify the size specific activity, but rather to demonstrate activity variations *within a set* of particles of the *same* size. When it comes to size dependence, we report on the transition between mass transport limited and kinetically limited reaction regimes, for which our results confirm that 5 particles are enough.

3. P14 in supporting information: this section demonstrates that the fluorescence signal is proportional to the fluorescein concentration in the reference channels, where the authors should add experiments or literatures to confirm it. Additionally, in Au decorated channels, the concentration value of each time point is derived from the fluorescence intensity, however, the fluorescence intensity along the channel is affected by many factors. Thus, the quantitative relationship between the fluorescence intensity and fluorescein concentration should be provided.

Our reply: We agree that this statement was based on a (very reasonable) assumption. To unambiguously demonstrate it, as requested by the Reviewer, we have now also carried out additional experiments that demonstrate a linear proportionality between fluorescence intensity and fluorescein concentration (Figure SI 12b) and refer to it in the main article in the following way:

…the fluorescence intensity profile, that is, the fluorescein concentration profile (Figure SI 12b),

4. Line 174-178, P7 in main text: the authors should add more explanations and literatures to prove that the reaction is mostly mass transport limited and never reach the surface reaction limited regime. In addition, the sentence "This can be understood as that we for the largest …" should be changed to "This can be understood as that for the largest …"

Our reply: We have changed our wording as suggested. However, when it comes to the suggestion to add references that would strengthen our statement that the largest patches always are in a mass transport limited regime, we disagree with the Reviewer. The reason is that the specific conditions at which mass transport limitations occur are a direct consequence of the applied experimental system and its specifics, like reactor dimensions, reactant concentrations, flow rates, etc. In other words, results from other studies cannot be directly applied to our system. We would rather argue (as we do in the text in detail), that we convincingly can derive the conclusion of complete mass transport limitation from our results for the specific system at hand. This is one of the main points and findings of our work.

5. Line225-228, P9 in main text: more literatures should be supplied to confirm the conclusion that the spread activity of individual gold nanoparticle for a certain size can be assigned to the different abundance of low coordination and defect sites imposed by the microstructure.

Our reply: We have added several references that confirm the general validity of our statement.

6. Line 269, P11 in main text: "increasing linearly from ca. 0.1 – 0.3 s-1 per site" should be "increasing linearly from ca. 0.01 – 0.03 s-1 per site" according to the Figure 4.

Our reply: We thank the reviewer for spotting this error – we have corrected accordingly.

7. There are two SEM images missing in Figure SI 4d.

Our reply: This is observed correctly – the reason being that these two particles could not be imaged due to contamination. We have made this clear in the caption of Figure SI 4d.

Reviewer #2

In their manuscript Levin et al. have demonstrated the use of nanofluidic device for parallel analysis of the catalytic reactivity of single nanoparticles in solution. The main advantages of the experimental setup presented by the authors are: 1. Simultaneous analysis of particles reactivity under identical reaction conditions and 2. Conducting reactivity measurements under realistic reaction conditions. The authors have studied with their setup the influence of particles' size on the reactivity under various reactants concentrations. They have identified that as the particles' size is increased beyond 100 nm, the reactivity is less influenced by changes in reactants concentrations and is less sensitive to variations in the surface properties of the nanoparticles.

The authors describe an interesting study about size-dependent reactivity, which they identified based on single nanoparticles measurements in a nanofluidic setup. However, this experimental approach leads to major limitations in the scientific knowledge that can be extracted, as detailed below:

1. The authors present a different approach for fluorescence microscopy measurements of catalytic reactions, which is in a way opposed to the mainstream approach. The common way to use fluorescence microscopy measurements is by probing the formation of fluorescent products and then by using super-resolution techniques, identifying the site-dependent reactivity with a resolution of < 10 nm (see for example, JACS 132 (1), 138-146 and ACIE 48 (49), 9285-9289). The high spatial resolution is achieved by using very low reactants concentration – thus enabling the detection of individual reactions and mapping site-dependent reactivity on single nanoparticles.

Our reply: The Reviewer is correct that our presented approach is different from the "mainstream approach" using super-resolution microscopy and we want to highlight that this also is one of the key points of our work. While we "sacrifice" the single molecule as well as high spatial resolution, we can instead operate our catalytic reaction at reactant concentrations that are more relevant from an application perspective, because it is well known that for example surface coverage of reactants on the catalyst may have a strong influence on its function.

The authors' approach for detection of catalytic reactions limit their spatial resolution to the diffraction limit of light (~ 200 nm), which is larger than most of the particles studied in this work.

Our reply: This is only partly correct because nothing precludes super-resolution imaging in our system technically speaking. However, in the present work we chose to not use this approach due to the required very low reactant concentrations. Instead, we want to shed light on other important aspects of catalysis at relevant reaction conditions and showcase how we at the single nanoparticle level can map the transition from mass transport control to surface reaction control in nanoconfinement, similar to what is found in nano-or mesopores of widely used catalyst support materials. Hence, this aspect is of importance from a chemical engineering perspective, for example. Secondly, since we do not investigate the particles themselves with the fluorescent readout it is not of importance that the particles are smaller than the spatial resolution of our experiment.

At this spatial resolution, the authors could have used micro-Raman measurements to map the catalytic transformation in their nanofluidic reactor with some payment in the spatial resolution (500 nm) but with a much richer chemical understanding of the catalytic reaction. Raman micro-spectroscopy measurements with CCD detectors can provide simultaneous detection of the reactivity on multiple nanoparticles.

Our reply: It is correct that micro-Raman measurements would provide richer chemical understanding, and, in principle, nothing technically prevents us from using Raman spectroscopy with our nanofluidic reactor system, other than the simple fact that we have no access to such instrumentation. Furthermore, the intensity-concentration relationship from SERS would presumably not scale linearly at the high concentrations employed in this work, making quantification a challenging task (Campion, A. and Kambhampati, P., Chem. Soc. Rev., 1998,27, 241-250. Stolberg, L., et al., J. Electroanal. Chem., 1991, 300, 563-584). Finally, as mentioned above, one of the key points of our work is that we can probe single nanoparticles at relevant reactant concentrations all the way from mass transport limitation to surface reaction limitation. Thus, we strongly feel that attempting such experiments with micro-Raman at the current stage are beyond the scope of this work and revision.

That said, we of course very much appreciate the suggestions made by the Reviewer and it is indeed part of our plans to employ the developed platform both in combination with, and as a complement to, super-resolution microscopy and Raman spectroscopy. To highlight these possibilities and discuss fluorescence in comparison with Raman, we have added the following sentences to the outlook section of our revised manuscript:

In comparison with the state-of-the-art single particle catalysis methods of SMFM (which give insights in the spatial position of the active sites due to superior spatial resolution) and SERS (which offers specific chemical information), our platform enables catalyst operation at technically relevant reactant concentrations and resolves reactant concentration gradients up- and downstream of single catalyst particles.

and

Furthermore, in principle, our nanofluidic reactor platform can be applied to SMFM, SERS and/or SPS readout to achieve a wider measurable concentration range and in this way compensate for some of the drawbacks of these techniques.

By tracking the transformation from a fluorescence reactant to a non-fluorescence product the authors have lost the benefits of super-resolution microscopy while using a probing technique that provides very limited molecular understanding of the reaction. Therefore, it is not clear to me that the model reaction and the probing technique (fluorescence microscopy) can effectively address the scientific question that the authors have raised.

Our reply: Indeed, we have sacrificed the benefits of super resolution microscopy but, as discussed in detail already above, are instead able to probe the catalyst at technically relevant reactant concentrations and over a wide regime that includes surface reaction limitation and mass transport limitation, imposed by nanoconfinement.

2. In their manuscript, the authors describe the current limitations in state-of-the-art florescence microscopy as: 1. low throughput and 2. low reactant concentrations and/or reaction rates. This statement is not accurate. Fluorescence microscopy measurements were previously performed on large scale, covering the reactivity of hundreds of nanoparticles (ACS Catal. 2013, 3, 1448-1453). The use of low reactants concentration was specifically chosen in fluorescence measurements in order to reach super-resolution conditions.

Our reply: In our manuscript, we describe the current limitations in the state-of-art single nanoparticle measurement techniques *in general* as low throughput or low reactant concentrations and/or reaction rates, not specifically for fluorescence microscopy. We are well aware that fluorescence single nanoparticle catalysis has been performed at large scale and are citing the article mentioned above in our manuscript already. As mentioned earlier, our approach sacrifices the super resolution to be able to reach higher concentrations needed to study transition from the reaction to the mass transport limited regime.

3. The authors have detected that as the size of the particles is increased there is less variation in the particles reactivity and the reactivity is not limited by surface-reaction. The authors have correlated this observation with differences in the density of low-coordinated surface sites. As I see it, this correlation needs to be further validated:

a. The authors show that the reactivity is widely changed once the particle size is increased from 64 to 100 nm. These particles are already very large (in comparison to highly reactive Au nanoparticles, which are in the size range of <10 nm). Why are there such vast differences in the reactivity of particles in this specific size range?

Our reply: The reason for this is *not* mainly to be found in the fact that the 64 nm particles have significantly more variation in activity than the 100 nm ones but rather that the 100 nm particles exhibit a surface area that is ca. two times larger, meaning that (assuming same activity per site on average) twice the amount of fluorescein reacts per unit time. This, in turn, means that the mass transport limited regime is retained up to higher concentrations for the larger particle, which – seemingly – gives rise to an *apparent* lower variation in activity. To make this clear, we have revised the following sentence in the text from

The observed decreased spread in activity between particles for increasing size can be attributed to a larger degree of mass transport control due to the limited reactant supply by the nanochannel.

to

The observed decreased spread in activity between particles for increasing size can also be attributed to a larger degree of mass transport limitation for larger particles due to the restricted reactant supply by the nanochannel. This restriction is evident for sizes 102-128 nm in Figure 3 but is not found across all experiments or when higher fluorescein concentrations are applied (Figures SI 19-21).

Are there any clear evidence for differences in the density of low coordinate surface atoms within the probed particles?

Our reply: Yes. As can be seen in Figure SI 2, there is large heterogeneity in the amount of crystal grains between the particles (0-10 grains per particle) resulting in a large variation in number of grain boundaries. Since a grain boundary is the interface between two crystallites giving rise to high abundance of defects and low coordination sites, we propose that the variation in number of grain boundaries can be assigned as a measure of variation in number of defects and low coordination surface atoms. To clarify this, we have added the following subordinate clause and references to the revised text

, containing copious low coordination sites due to lattice orientation mismatch between crystallites.

to the sentence.

This becomes apparent from the TEM images of a large set of representative particles that exhibit widely varying structure and abundance of highly defectuous grain boundaries.

I would assume that annealing (500-600 C) of the smaller particles would result a decrease in their density of low-coordinate surface sites – was this experiment done? What is the influence of such pretreatment on the reactivity of the various nanoparticles?

Our reply: The main source of low-coordination sites in our particles are grain boundaries and these prevail at the applied annealing conditions.

b. The authors have shown that during the preparation of Au particles there were few smaller particles that were formed and were washed away by various pretreatments. Did the authors conduct HR-SEM measurements to identify that there are no small (> 10 nm), highly reactive Au nanoparticles on the surface?

Our reply: We have not done any HR-SEM measurements but instead used TEM measurements to indeed show that the <10nm particles disappear, as summarized in Figure SI 3.

Did the authors check for any leaching and presence of Au residues in the products solution?

Our reply: This is a valid comment that we also have thought about. However, estimating the possible concentration from the *total* amount of Au for all nanoparticles in a chip in a small ICP-MS sample volume of 5 ml results in an Au concentration of ~0.1 ng/L. This is at the limit of what can be detected with ICP-MS and, therefore, any leaching residues would be impossible to detect.

Since this question, however, is really important, we applied a different approach by performing HRTEM-EDS scans across the edge and in the surrounding of a single nanoparticle after three cleaning cycles. In these experiments we did not detect any trace of Au on the surface near the nanoparticle and thus conclude that leaching can be excluded. This new data is added in the supplementary information (page 6 and Figure SI 3).

c. The authors use the fluorescence quenching as a marker for reactant-to-product transformation. Additional experiments are needed in order to molecularly identify the product and verify that the non-fluorescence product is the major/only product that is formed in the reaction.

Our reply: The product, the reduced form of fluorescein, has been isolated and characterized with NMR, HRMS and IR. The corresponding information of how this was done is presented in the supplementary information where also the spectral data can be found (section 3 and Figure SI 9).

d. The authors have shown that for small particles as the fluorescence reactant concentration is increased the reactivity is decreased. This indicates that the reaction is controlled by Langmuir-Hinshelwood mechanism and not by Eley–Rideal mechanism. The authors should verify that experimentally, by for example changing simultaneously the concentration of the two reactants.

Our reply: To increase our understanding of the reaction mechanism we performed several batch reactions using colloidal gold nanoparticles and varied reactant concentrations in the ranges 0.3 µM to 10 mM for fluorescein and 5 to 200 mM for sodium borohydride. However, in all measurements, the reaction rates consequently follow first order relations to both fluorescein and borohydride or are too slow to be effectively measured. These results neither contradict or confirm the Langmuir-Hinshelwood or the Eley-Rideal mechanism. Based on our previous measurements done in the nanofluidic channels however (Figure 3 and SI 19-21), the decreased reactivity at higher fluorescein concentrations speaks against an Eley-Rideal mechanism since, for the Eley-Rideal mechanism, there is no competition for binding sites which could account for this decrease. Nevertheless, since we still have no explicit further proof for the reaction being of Langmuir-Hinshelwood type, we have added a sentence (in italics below) to the text to indicate that we propose a Langmuir-Hinshelwood reaction on a speculative rather than absolute basis:

The reaction rate is then governed by the interplay between both reactant concentrations and adsorption and desorption constants, which means that when the surface concentration of fluorescein becomes larger than the surface concentration of borohydride the ToF will decrease due to “poisoning” of the Au surface by fluorescein. However, the ToF dependence found for our system shows a slightly steeper decrease at higher fluorescein concentrations than predicted purely by a Langmuir-Hinshelwood mechanism (Figure SI 25*). This could either indicate that the reaction is not of the Langmuir-Hinshelwood type or that the reaction step is not rate determining, which would render the approximation invalid.* Regardless, it is most certainly mainly a consequence of the tiny volume of the nanochannel (8.75µm^3 for the entire channel), which accelerates the transition between the mass transport and the surface reaction limited regimes, since when most of the molecules passing the particle also react, the limited mass transport will lead to a lower *local* reactant concentration in solution.

Reviewer #3

This research article, by Langhammer, Westerlund and co-workers, reports on the development of a nanofluidic device, which in combination with fluorescence microscopy allows to investigate metal nanoparticles in various shapes ands sizes to assess the interparticle heterogeneities in terms of catalyst activity. For this purpose, the authors have selected the already explored catalytic reduction of fluorescein by borohydride on Au nanoparticles. One of the conclusions is that the setup allows to investigate the TOF of the Au nanoparticles and this number is in the range of 0.1 s-1 per site.

The strength of the paper is its catalyst chemical engineering aspects, in which the authors explore the regimes between diffusion/mass transport control up to surface reactivity control; hence they use their new device to explore what it can learn in real-life catalytic applications.

Our reply: We appreciate the Reviewer's positive assessment to our experimental approach.

However, on the other hand, one has to recognize that the reactions proposed are father exotic and far away of a real life catalytic system (in terms of metal nanoparticle size; as well as reaction conditions and reaction system). That is the weak part; as one of course wish to see how it can be applied in a broader sense.

Our reply: This is an interesting point that in our opinion raises the issue of the relevance of model catalysts and model reactions in general, where one of course always can argue that such systems are not relevant for "real" catalysis. While this on one hand may be correct, it is also indisputably true that such experiments have provided invaluable insights to the understanding of catalytic processes. It is therefore on the background of this "dilemma" that we would like to phrase our response.

Our study presented here is to 100% a model system type work where we indeed use nanoparticles that are larger than "real" catalyst and a reaction that may not be very relevant industrially. We do this to demonstrate the concept. At the same time, and this is the key point that we may have failed to highlight in the first version of our work, there are no limitations to, for instance, the particle size. Using electron-beam lithography methods, for instance, it is possible to make nanoparticles as small as 5 nm, which would be in the relevant range of "real" catalysts (Grigorescu, A. E., *et al.* Nanotechnology 2009, 20, 292001, Manfrinatio, V.R., *et al.* Nano Lett 2013, 10, 1555-1558.). Furthermore, already to date, and as part of a forthcoming study, we are able to place individual shape and size-selected colloidal nanocrystals in these devices, one per nanochannel, as shown in the figure below. Therefore, we feel it is fair to say that our experimental platform is relevant from a "real life" catalysis perspective.

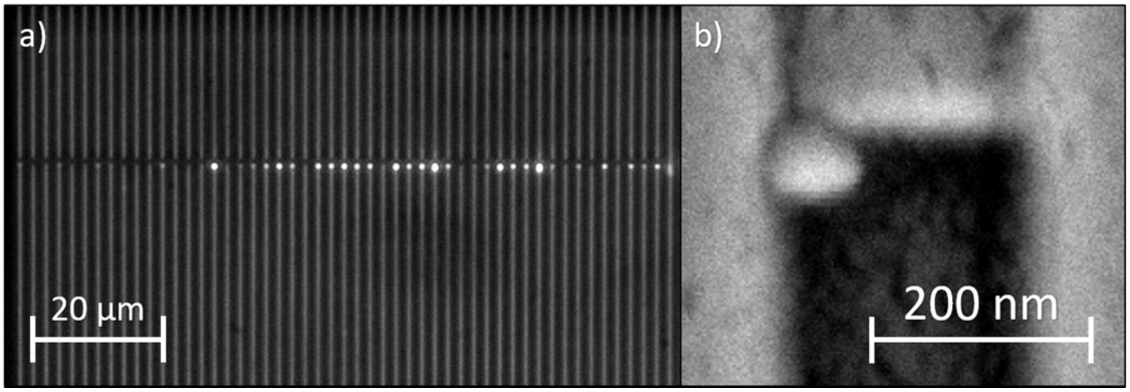

Colloidal particles placed in nanofluidic traps. a) Dark field scattering image of colloidal particles of 60 nm in size stuck in a trap the nanofluidic channels. b) SEM image of a single colloidal nanoparticle in the nanofluidic trap.

When it comes to reaction conditions, we feel that the demonstrated fact that we can study one and the same single nanoparticle both at surface reaction-controlled conditions and under mass transport limitation is demonstration enough that conditions can be varied very widely. Finally, when it comes to reaction system, of course any experimental approach that relies on fluorescence will be significantly limited to reactions that either produce or quench fluorescence. Nevertheless, in the context of model reactions/systems it has been widely proven to be a very powerful concept to shed light on catalytic reactions. Furthermore, as a step beyond what is already discussed above in response to Reviewer #2, there are no technical limitations that prevent application of our platform to Raman readout to (i) relax the boundary condition of fluorescence and (ii) obtain more chemical information. Presently, however, we do not have access to a suitable micro-Raman system, which is why we are unable to explicitly demonstrate this. Nevertheless, to explicitly mention these future opportunities, we have added the following text to the outlook session:

Looking forward, we predict that our platform will not only be usable in combination with traditional fluorescence microscopy readout but also with super-resolution microscopy techniques and with micro-Raman spectroscopy to enable deeper chemical insights. Furthermore, either by utilizing nanofabrication for particles in the sub-10 nm size range (Grigorescu, A. E., *et al.* Nanotechnology 2009, 20, 292001. V.R., M., *et al.* Nano Lett 2013, 10, 1555-1558.) or by specifically placing individual colloidal nanocrystals inside nanofluidic channels, it will become possible to extend the approach down into a size or structure range directly relevant for “real” catalysis beyond model systems.

What I also miss is the comparison with e.g. Raman/SERS type approaches for monitoring in nanofluidic devices the reaction processes. This offers insight in the reaction course as the reaction products and reactants and intermediates can be assessed via the spectra; what is not possible with the fluorescence microscopy approach (or at least not applied in this system).

Our reply: Here we refer both to our response on the same topic to Reviewer #2 and the discussion above and highlight again that we at this point have no possibility to execute micro-SERS experiments.

I am charmed by the mathematical approaches taken in this work; that is the strength of the work. An example being Figure 2c; here one can also see what is possible and that is evident from figure 3. Figure 5 is in my opinion not very clear and the text surrounding this figure can be better written.

Our reply: We are happy that the Reviewer appreciates our modeling approaches and the insights they provide. Following his/her advice, we have modified the text related to Figure 5 in the following way:

1. Clarified figure caption from

b) ToF with subtracted mean value for each particle size obtained from a total of nine independent experiments carried out on the same sample (chip 1). The gray area depicts the span between the highest and lowest ToF values for each particle, the dotted line corresponds to the mean value of each particle for all nine measurements and the colored dots indicate the specific values of ToF with subtracted mean obtained for each particle in each measurement.

to

b) ToF with subtracted mean value for each particle obtained from a total of nine independent experiments (such as displayed in Figure 3 and Figures SI 19-21) carried out on the same sample (chip 1). The colored dots indicate the specific values of ToF with subtracted mean obtained for each particle in each measurement, the gray area depicts the span between the highest and lowest values and the dotted line corresponds to the mean value of each particle for all nine measurements.

2. Added more description to the correlation coefficient from:

Furthermore, to also address this in a mathematically robust way, we calculated the correlation coefficients between trends in maximum ToFs with subtracted mean (inset Figure 5b and Figure SI 28).

to

Furthermore, to also address this in a mathematically robust way: we calculated the correlation coefficients ($\frac{Cov(f,g)}{\sigma_f \cdot \sigma_g}$, where f and g are measurement series values and σ_i are their standard deviation), between ToFs with subtracted mean for both chips (inset Figure 5b and Figure SI 28).

The concept of single particle catalysis should also be better explained and stressed.

Our reply: We have added the following sentence to our introduction:

Therefore, measurement on single nanoparticles are essential to uncover particle specific properties.

As a side remark, as pointed on page 2, the work described is not operand but in situ work.

Our reply: We have reworded accordingly

I also not that the references are not well written; e.g. ref. 5, article number is missing; ref. 32, reference not correctly written; and ref. 28, reference not correctly written; but there are more errors like this.

Our reply: We have amended the missing article number of ref. 5. However, we do not see anything wrong with ref 28 and 32 and have looked over all references to have them according to the standard of Nature Communications.

What I miss in the paper is the general applicability (as mentioned already above) and would here like to see an expansion to another reaction and/or another catalyst system; to show what can be done with this nano-fluidic device next to what the authors already discuss. Currently, it remains a showcase for what can be done, but not really much new catalytic chemistry has been learned.

Our reply: To demonstrate our nanofluidic reactor platform for a second reaction, we have executed a preliminary study of amplex ultra red oxidation to resorufin by hydrogen peroxide over our Au nanoparticle catalyst and included the main findings in a new section 7 in the Supplementary Information. In the main text, we refer to this new section in the following way:

and to other types of reactions where a fluorescent product is formed, as demonstrated in a preliminary fashion in SI section 7 (Figures SI 29-30) for the catalytic oxidation of amplex ultra red to resorufin over an Au nanoparticle catalyst.

Again, it is a very nice chemical engineering approach to the concepts of single particle catalysis and the authors have devised and constructed a nice setup, however, illustrating it potential with a rather well known, and in some way a boring chemical reduction reaction.

Our reply: This was done on purpose because only in this way we can benchmark our approach. It is part of forthcoming works to apply our platform to less-characterized reactions.

Having said all this, the work is to the best of my knowledge novel, and also deserves to be published in a journal such as this one; hence I recommend publication after revisions as outlined above.

Our reply: We appreciate this assessment in terms of novelty and in general the very constructive comments provided by Reviewer #3.

REVIEWERS' COMMENTS:

Reviewer #2 (Remarks to the Author):

In the revised version of their manuscript titled "A nanofluidic device for parallel single nanoparticle catalysis in solution" the authors have been able to address most of the concerns that were raised by this reviewer.
Specifically, the authors have performed few additional measurements in order to clarify that there is no leaching of Au atoms or presence of highly-reactive small Au nanoparticles in the nanochannels.
The authors have performed additional measurements to identify the mechanism of the reaction (though these experiments did not lead to a solid conclusion about the reaction kinetics).
Importantly, the authors have provided a more comprehensive discussion about the pros-and-cons of their experimental approach.
Therefore, as this reviewer sees it, this manuscript can be now accepted for publication in Nature Communications.

Reviewer #3 (Remarks to the Author):

I have read the rebuttal letter (as referee 3) and noted the comments of the authors to the three referee reports, including the comments I have raised on the initial comments. I regret to see that the authors did not do a lot with my comments; l and had hoped that they would make an effort to include a less exotic reaction than currently reported in the paper. On the other hand, conceptually the paper is providing a new approach, which will inspire research groups in the (near) future to evaluate the concepts provided. I also have noted (again) that the paper is a nice addition to the literature and as such is also a nice piece of research, worthwhile to be published in a general science journal, such as Nature Communications. With this in mind, and also noting the answers to the other two referee reports, I am happy to recommend this work for publication in this journal.
